# Spatio-temporal characterization of phenotypic resistance in malaria vector species

**DOI:** 10.1186/s12915-024-01915-z

**Published:** 2024-05-20

**Authors:** Eric Ali Ibrahim, Mark Wamalwa, John Odindi, Henri E. Z. Tonnang

**Affiliations:** 1 International Centre of Insect Physiology and Ecology (Icipe), PO box, Nairobi, 30772 Kenya; 2 School of Agricultural, Earth, and Environmental Sciences, University of KwaZulu-Natal, Pietermaritzburg, 3209 South Africa

**Keywords:** Cellular automata, Insecticide resistance, Malaria vectors, Spatio-temporal modeling

## Abstract

**Background:**

Malaria, a deadly disease caused by *Plasmodium protozoa* parasite and transmitted through bites of infected female *Anopheles* mosquitoes, remains a significant public health challenge in sub-Saharan Africa. Efforts to eliminate malaria have increasingly focused on vector control using insecticides. However, the emergence of insecticide resistance (IR) in malaria vectors pose a formidable obstacle, and the current IR mapping models remain static, relying on fixed coefficients. This study introduces a dynamic spatio-temporal approach to characterize phenotypic resistance in *Anopheles gambiae* complex and *Anopheles arabiensis*. We developed a cellular automata (CA) model and applied it to data collected from Ethiopia, Nigeria, Cameroon, Chad, and Burkina Faso. The data encompasses georeferenced records detailing IR levels in mosquito vector populations across various classes of insecticides. In characterizing the dynamic patterns of confirmed resistance, we identified key driving factors through correlation analysis, chi-square tests, and extensive literature review.

**Results:**

The CA model demonstrated robustness in capturing the spatio-temporal dynamics of confirmed IR states in the vector populations. In our model, the key driving factors included insecticide usage, agricultural activities, human population density, Land Use and Land Cover (LULC) characteristics, and environmental variables.

**Conclusions:**

The CA model developed offers a robust tool for countries that have limited data on confirmed IR in malaria vectors. The embrace of a dynamical modeling approach and accounting for evolving conditions and influences, contribute to deeper understanding of IR dynamics, and can inform effective strategies for malaria vector control, and prevention in regions facing this critical health challenge.

**Supplementary Information:**

The online version contains supplementary material available at 10.1186/s12915-024-01915-z.

## Background

Malaria, caused by the protozoa parasite of the genus *Plasmodium* and transmitted to people through bites of infected female *Anopheles* mosquitoes, remains a significant global health challenge. In 2020 for instance, approximately half of the world’s population faced malaria risk, with 227 and 241 million cases reported in 2019 and 2020, respectively [1]. Over the same period, an estimated 627,000 and 558,000 malaria-related deaths occurred. Of the deaths reported, 95% occurred in sub-Saharan Africa, with children under 5 years accounting for 80% of these deaths [1]. Due to the high prevalence of malaria cases in Africa, the World Health Organization (WHO) African region carries a disproportionately high share of the global malaria burden. Notably, in 2020, four African countries Nigeria (31.9%), the Democratic Republic of the Congo (13.2%), Tanzania (4.1%), and Mozambique (3.8%) accounted for over half of all malaria deaths worldwide [1].

To combat malaria, several goals that include those outlined in the WHO Global Technical Strategy for Malaria 2016–2030 have been initiated. These goals, to be achieved by 2030, encompass (i) reducing malaria incidences by at least 90%; (ii) reducing malaria mortality rates by at least 90%; (iii) eliminating malaria in at least 35 countries; and (iv) preventing a resurgence of malaria in all countries that are malaria-free [1]. To realize these ambitious goals, the focus has shifted toward malaria prevention tools and strategies, which have significantly reduced the global malaria burden over the past two decades [1]. However, recent years have witnessed a mitigation slowdown or stagnation, particularly in highly burdened countries of sub-Saharan Africa [1, 2].

Methods that include malaria vaccine, preventive chemotherapies, and mosquito vector control are commonly used to control malaria. Mosquito vector control strategies encompass vectors genetic engineering and the application of insecticides [3]. However, the development of insecticide resistance (IR) among the mosquito vectors, and the spatial IR spread remains a significant challenge in using insecticides [1, 4]. Hence, over 78 countries have reported mosquito vector resistance to at least one of the four commonly used insecticide classes (pyrethroid, organochlorine, carbamate, and organophosphate) between 2010 and 2019 [1]. Additionally, 29 countries have reported mosquito vector resistance to the four major insecticide classes [1]. Spatial distribution maps depicting IR reveal alarming prevalence, particularly for pyrethroids and dichlorodiphenyltrichloroethane (DDT) across sub-Saharan Africa from 2005 to 2017. In some areas, the vectors’ mean mortality following insecticide exposure declined from almost 100% to less than 30% [5].

Insecticide resistance (IR) can occur through various mechanisms broadly categorized into four types: target-site, metabolic, behavioral, and cuticular resistance. Target-site resistance is primarily caused by mutations at the site of action of an insecticide, which subsequently reduces or prevents the insecticide’s binding affinity [6–8]. Metabolic resistance arises from increased detoxification resulting from over-expression or conformational changes of the enzymes involved in the metabolism, sequestration, and excretion of the insecticide [7, 9]. Behavioral resistance involves any modification in the insect’s behavior that helps it avoid the lethal effects of insecticides [8, 10], while cuticular resistance arises from reduced uptake of insecticide because of the modifications in the insect cuticle [6, 7, 11].

For effective vector control, comprehensive information on the occurrence, extent, and temporal dynamics is necessary [5]. To acquire such critical insights, an increasing number of studies have focused on susceptibility tests and the modeling and mapping of the spatial distribution of IR in malaria vectors. The spatial modeling techniques commonly employed include Bayesian geostatistical, generalized linear models (GLMs), generalized additive models (GAMs), and mapping using geographic information system (GIS) [5, 12, 13]. Several studies have also employed mathematical models to elucidate the population dynamics, and evolution of IR in vector populations. Notably, study by McCormack et al. [14] utilized a stochastic metapopulation model to explore mosquito population dynamics emphasizing the effects of breeding site fragmentation on fine-scale mosquito population dynamics. Yamashita et al. [15] introduced a numerical model to simulate the population dynamics of Aedes aegypti in an urban neighborhood. The study involved use of finite volume method, and integrated various external factors such as wind patterns, chemical insecticide usage, and topology data to understand mosquito population spread within the city. Another study by da Silva [16] explored the impact of releasing genetically modified male mosquitoes on the spatial dynamics of Aedes aegypti populations. Further contributions to the field include the development of a deterministic mathematical model focusing on mosquito population dynamics with chemical control interventions [17], and the integration of mathematical models with trap data to estimate the growth, prevalence, and management strategies for Aedes aegypti [18]. Other mathematical modeling efforts have aimed at understanding mosquito population dynamics [19], dispersal patterns in varied environments [20], and the effects of seasonal changes on mosquito population sizes [21].

The accuracy and robustness of the models employed are intrinsically tied to the quality of the gathered data. However, modeling efforts are confronted by numerous challenges, chief among them being the scarcity of IR data, particularly in regions where susceptibility tests have not been conducted. Furthermore, mapping IR involves navigating diverse agro-ecological zones, each with its unique physical characteristics, vector habitats, ecological nuances, and vector biological traits. These agro-ecological variations introduce complexity into the modeling process, as the IR dynamics may vary significantly across different zones. Hence, understanding the intricacies of vector ecology and their responses to insecticides within these various ecological contexts is essential for the development of robust and accurate models. To develop an optimal model, it is essential to account for the variability in IR attributed to different ecological zones. Whereas current models provide valuable insights, they are static in nature, meaning that the coefficients estimating the level of IR remain constant over time. However, the spread of IR is akin to a diffusion process, influenced by a cause-and-effect mechanism. Hence, it is necessary to consider the adoption of dynamic models as they have the capability to capture the spatial and temporal dynamics of confirmed IR in various locations. Unlike static models, dynamic models recognize that IR levels can change over time due to evolving ecological conditions and selective pressures. However, to the best of our knowledge, there has been no study that utilizes a dynamic model to characterize IR. We believe that such an approach could provide a new perspective and a more accurate representation of the evolving nature of IR, especially in the context of changing ecological conditions and the complex cause-and-effect mechanisms at play. In this study, we introduce an innovative model designed to characterize the spatio-temporal phenotypic resistance in the *Anopheles gambiae* complex and *Anopheles arabiensis* malaria vector species.

## Results

### Exploratory data analysis

Principal component analysis (PCA) results for *An. gambiae* complex and *An. arabiensis* indicate that the first eight (8) principal components (PCs) explain over 80% of the variation in the data for pyrethroid, organochlorine, and organophosphate classes. All PCs have eigenvalues greater than 1, which is consistent with the Kaiser criterion suggesting the use of PCs with eigenvalues exceeding 1. The summarized results are presented in Table 1.

**Table 1:** Principal component analysis (PCA) results for *Anopheles gambiae* complex and *Anopheles arabiensis* for pyrethroid, organochlorine, and organophosphate classes

		*Anopheles gambiae* complex	*Anopheles arabiensis*
Insecticide class		**PC8**	**PC6**
Pyrethroid	Eigenvalue	1.035	1.301
	Proportion of variance	0.023	0.038
	Cumulative proportion	0.810	0.815
Organochlorine		**PC8**	**PC5**
	Standard deviation	1.036	1.410
	Proportion of variance	0.023	0.046
	Cumulative proportion	0.810	0.818
Carbamate		**PC19**	**PC9**
	Eigenvalue	0.960	1.226
	Proportion of variance	0.020	0.042
	Cumulative proportion	0.804	0.830
Organophosphate		**PC8**	**PC8**
	Eigenvalue	1.020	1.311
	Proportion of variance	0.023	0.048
	Cumulative proportion	0.805	0.841

*PC* principal components

For each PC, the drivers considered important (i.e., those with absolute correlation coefficients of > 0.2) are listed as supplementary materials (Additional file 1: Table S1). The results showed that a majority of PC1 consisted of drivers related to agricultural activities.

In the correlation analysis, it was observed that IR drivers were significantly correlated with confirmed resistance to different classes of insecticides. These drivers included human-related activities and characteristics such as crop farming and population densities and counts. However, the correlation tests also revealed weak correlations between most IR drivers and confirmed IR (Additional file 2: Table S2).

The cluster analysis was performed to identify groups of IR drivers that exhibited similar patterns. Additional file 3 presents both dendrogram and tabular summary of the clustering of drivers. These clusters were valuable in determining the similarity between variables, which was further supported by the correlation output results, and whether they explained the variation in the same component in PCA analysis.

The results of chi-square tests indicated a significant association between different LULC classes and IR states. The classes include urban and built-up lands, croplands, cropland/ natural vegetation mosaics, woody savannas, mixed forests, grasslands, barren, water bodies, savannas, permanent wetlands, evergreen broadleaf forests, closed shrub lands, open shrub lands, and deciduous broadleaf forest. Further, the use of indoor residual spraying (IRS) was found to be significantly associated with the IR states in most of the cases. Table 2 and Additional file 4 presents the results of chi-squared test.

**Table 2:** Results of chi-square test on the association between potential insecticide resistance (IR) drivers and the confirmed resistance state in malaria species

Species	Potential IR drivers	Insecticide class	Chi-squared value	df	*P*-value
*Anopheles gambiae* complex	Land use land cover	Pyrethroid	174.7500	26	< 0.0001
	Land use land cover	Organochlorine	104.7500	26	< 0.0001
	IRS pyrethroid	Pyrethroid	109.5300	2	< 0.0001
	IRS Organochlorine	Organochlorine	60.6070	2	< 0.0001
	IRS Carbamate	Carbamate	4.4636	2	0.1073
	IRS Organochlorine	Organophosphate	25.9680	2	< 0.0001
*Anopheles arabiensis*	Land use land cover	Pyrethroid	179.5200	28	< 0.0001
	Land use land cover	Organochlorine	112.1900	28	< 0.0001
	IRS pyrethroid	Pyrethroid	3.1051	2	0.2117
	IRS Organochlorine	Organochlorine	39.9720	2	< 0.0001
	IRS Carbamate	Carbamate	5.9679	2	0.0506
	IRS Organochlorine	Organophosphate	5.5849	2	0.0613

*df* degree of freedom, *IRS* indoor residual spaying, *IR* insecticide resistance

**Fig. 1:**
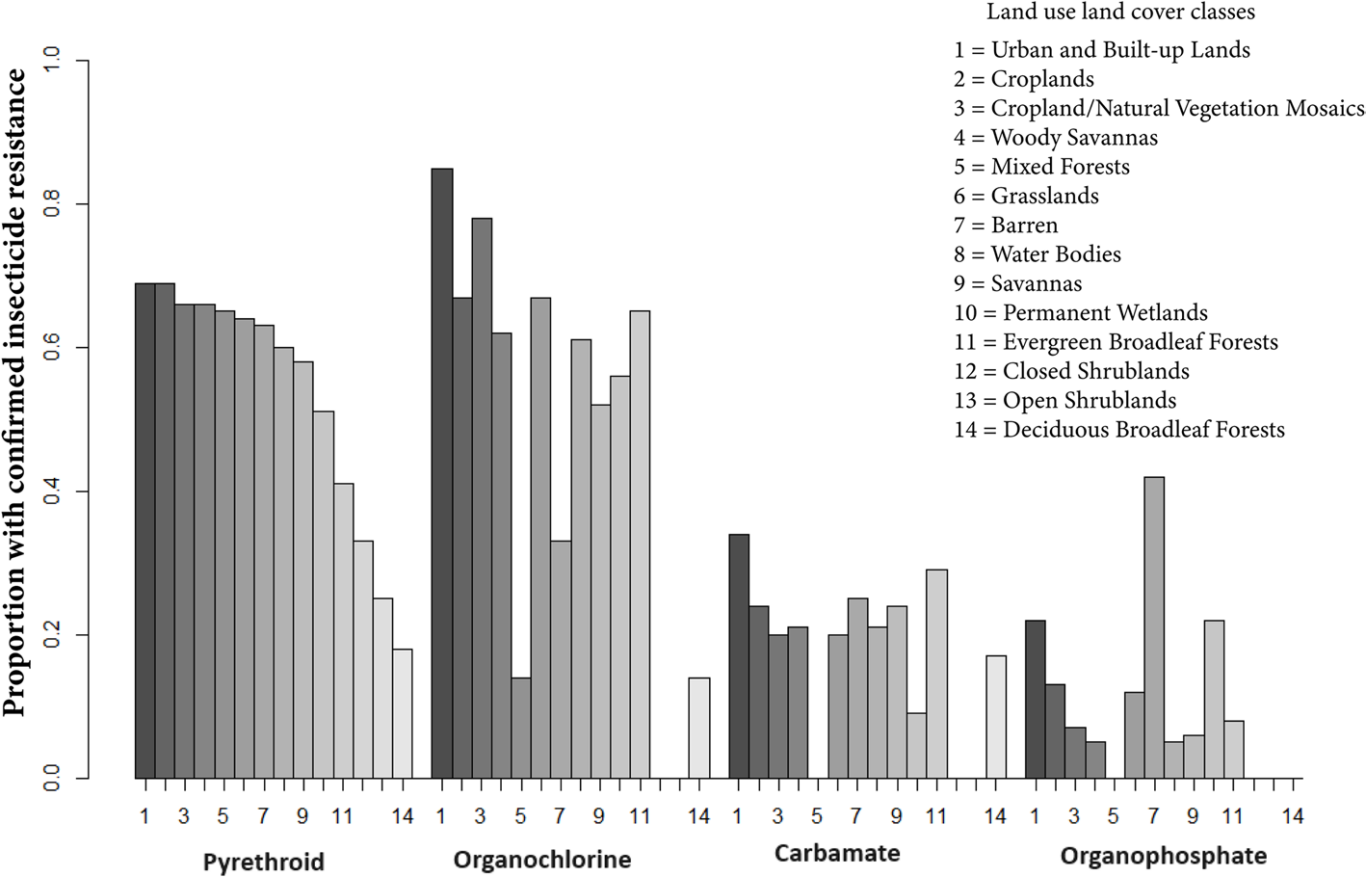
Distribution of confirmed insecticide resistance across various land use land cover classes

Within the LULC classes, urban and built-up lands, croplands, cropland/natural vegetation mosaics and grasslands, woody savannas, and water bodies had higher reported incidences of confirmed IR to pyrethroid and organochlorine. Further, barren land and mixed forests exhibited strong association with confirmed vector’s resistance to pyrethroids (Fig. 1).

### Spatio-temporal distribution of confirmed IR state in anopheles gambiae complex

**Fig. 2:**
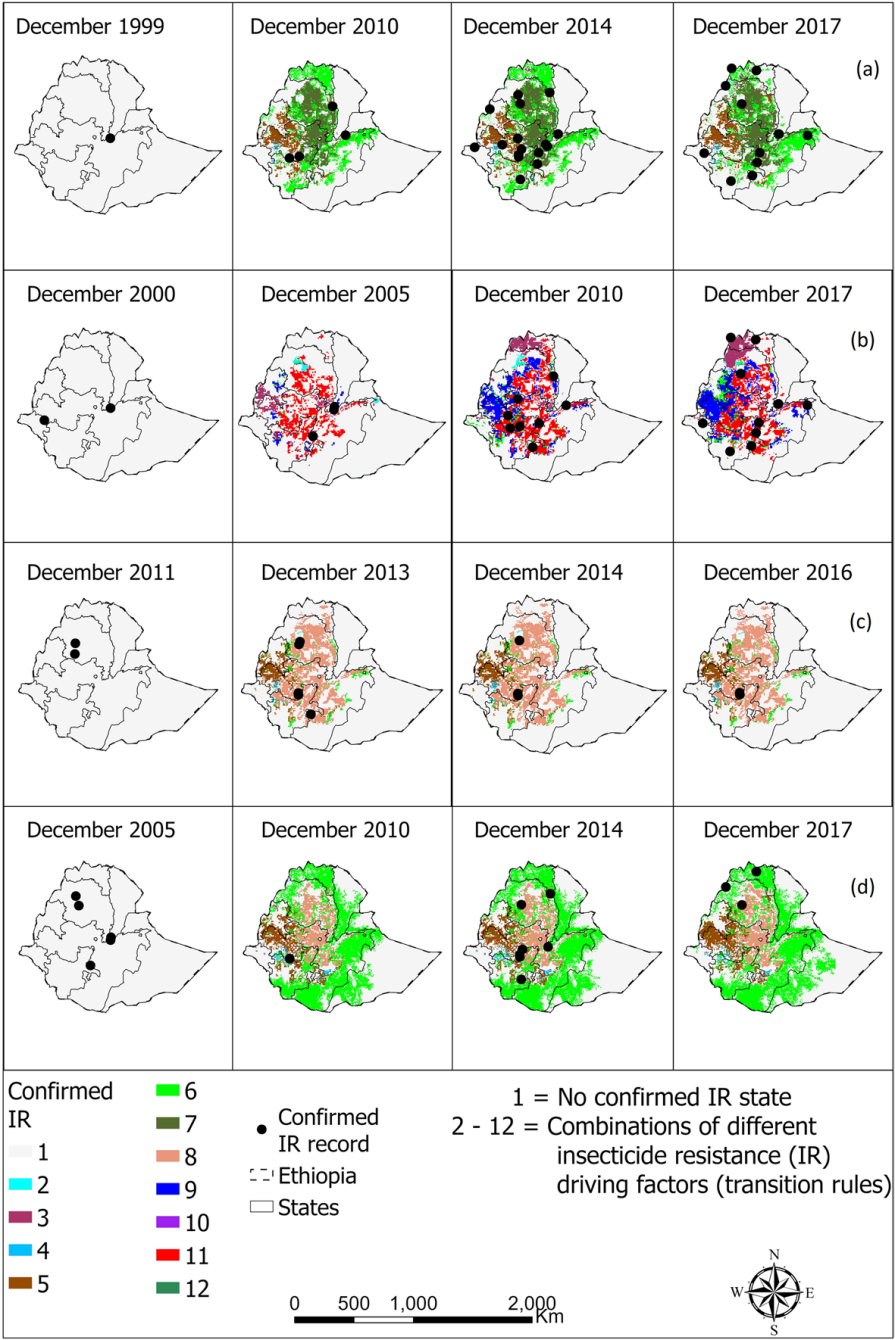
Spatio-temporal dynamics of confirmed resistance in *Anopheles gambiae* complex to **a** pyrethroid, **b** organochlorine, **c** carbamate, and **d** organophosphate in Ethiopia

**Fig. 3:**
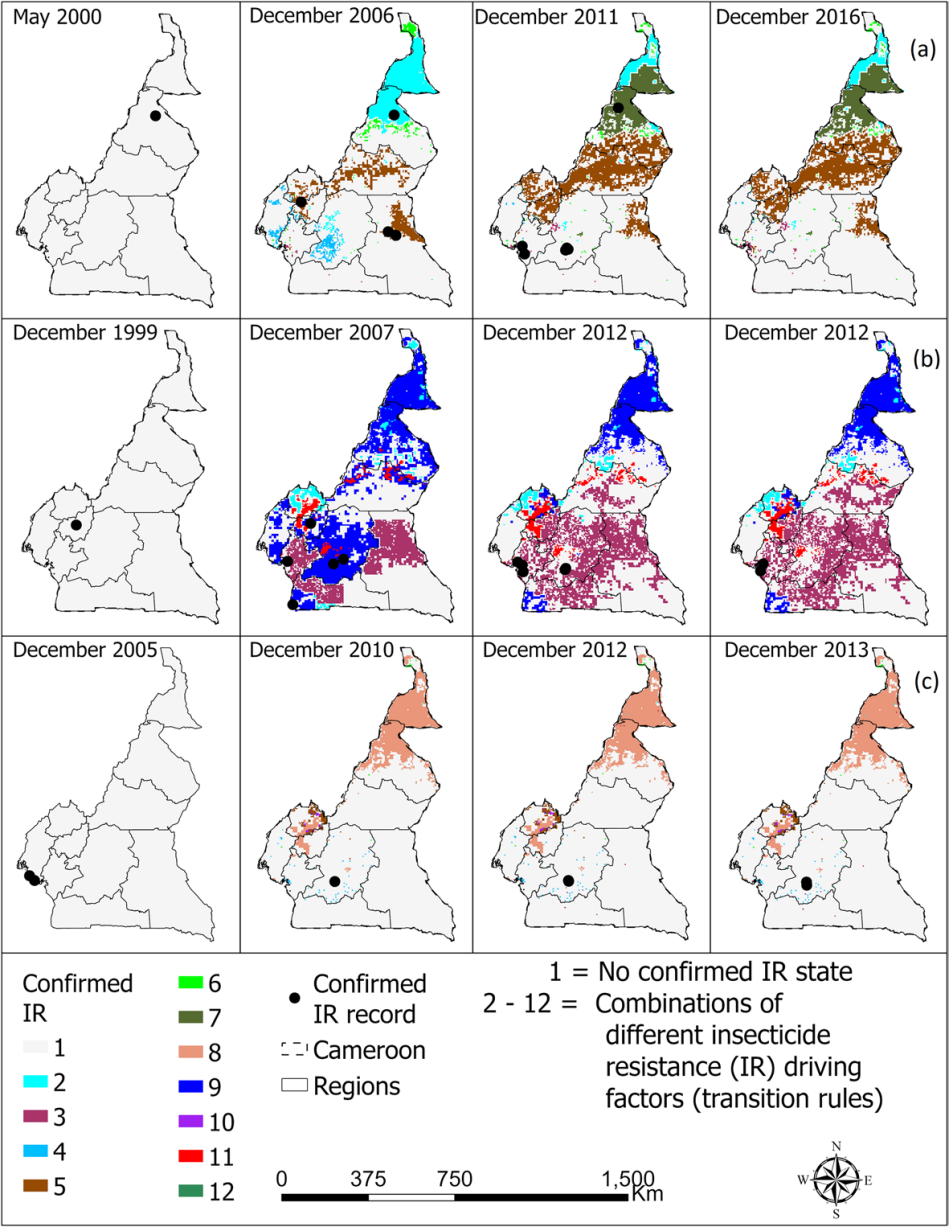
Spatio-temporal distribution of confirmed resistance in *Anopheles gambiae* complex to **a** pyrethroid, **b** organochlorine, and **c** carbamate in Cameroon

**Fig. 4:**
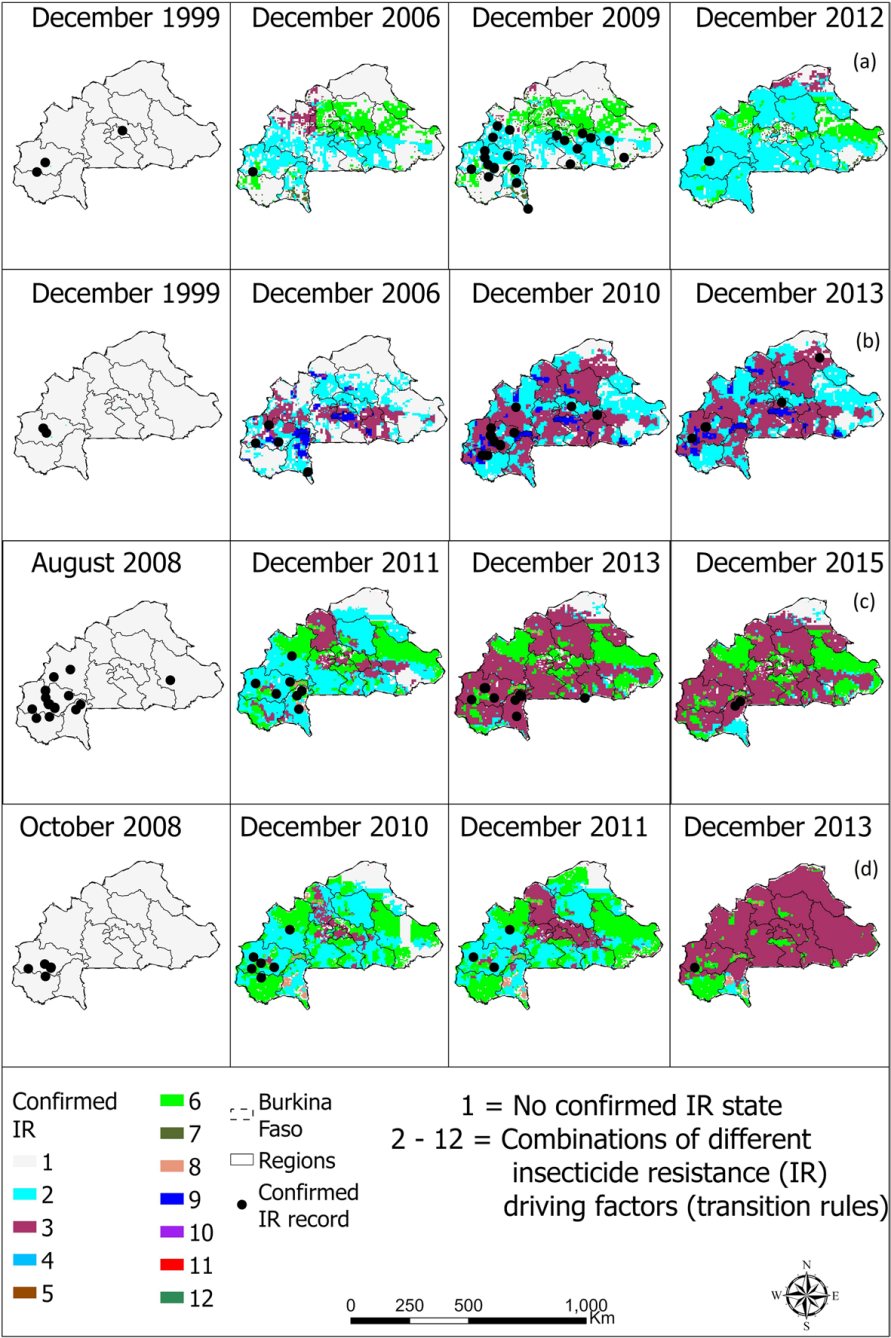
Spatio-temporal distribution of confirmed resistance in *Anopheles gambiae* complex to **a** pyrethroid, **b** organochlorine, **c** carbamate, and **d** organophosphate in Burkina Faso

The CA model successfully predicted the spatio-temporal distribution of confirmed IR in *An. gambiae* complex in Ethiopia, Cameroon, and Burkina Faso with accuracies over 80% in most cases after fine-tuning (Additional file 5: Table S4). Moreover, a significant proportion of the georeferenced points on confirmed presence of IR state coincided with the locations identified by the CA models as having a likelihood of confirmed IR state (Figs. 2, 3, and 4). Additional file 5 presents the accuracies obtained following comparison of the actual and predicted spatio-temporal distribution of confirmed IR in *An. gambiae* complex in different countries for the entire study period. However, Figs. 2, 3, and 4 present visual representation of selected years within the study period.

### Cellular automata (CA) model validation

**Fig. 5:**
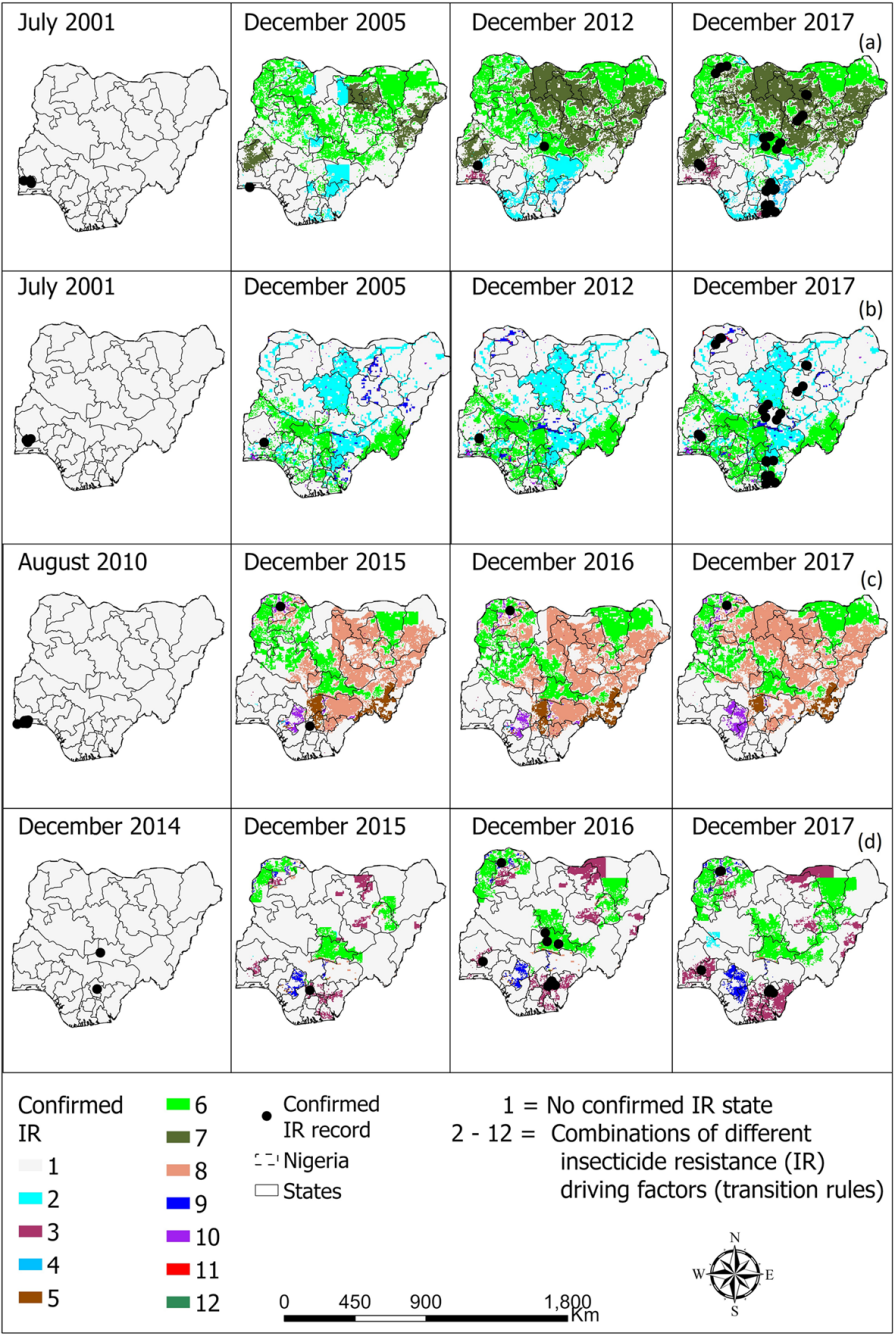
Spatio-temporal distribution of vectors confirmed resistance in *Anopheles gambiae* complex to **a** pyrethroids, **b** organochlorine, **c** carbamate, and **d** organophosphate in Nigeria

**Fig. 6:**
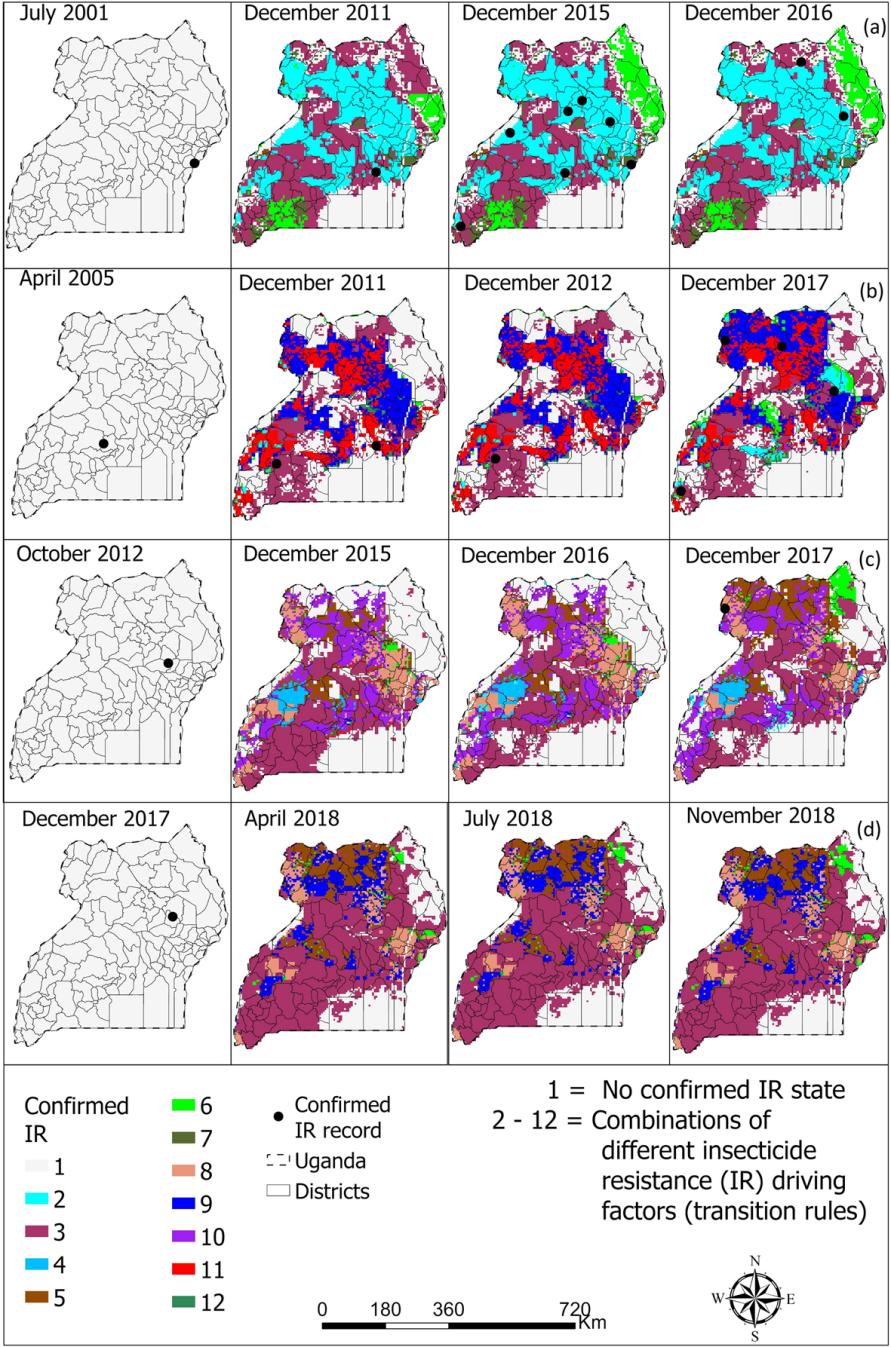
Spatio-temporal distribution of vectors confirmed resistance in *Anopheles gambiae* complex to **a** pyrethroid, **b** organochlorine, **c** carbamate, and **d** organophosphate in Uganda

The CA models, when validated in Nigeria and Uganda, accurately predicted the spatio-temporal dynamics of confirmed IR in *An. gambiae* complex with high accuracies over 80% in most of the years (Additional file 2: Table S2). When comparing the locations with reported confirmed IR state and the model’s predictions, it was observed that the majority of the actual and predicted confirmed IR locations coincided. Figures 5 and 6 provide visual representations of the model outputs for selected years, following model validation. The years present were selected because of high frequencies of reported confirmed IR compared to the excluded years. Additional file 2 presents the corresponding accuracy values for all the entire study period.

### Spatio-temporal distribution of confirmed IR state in Anopheles arabiensis

**Fig. 7:**
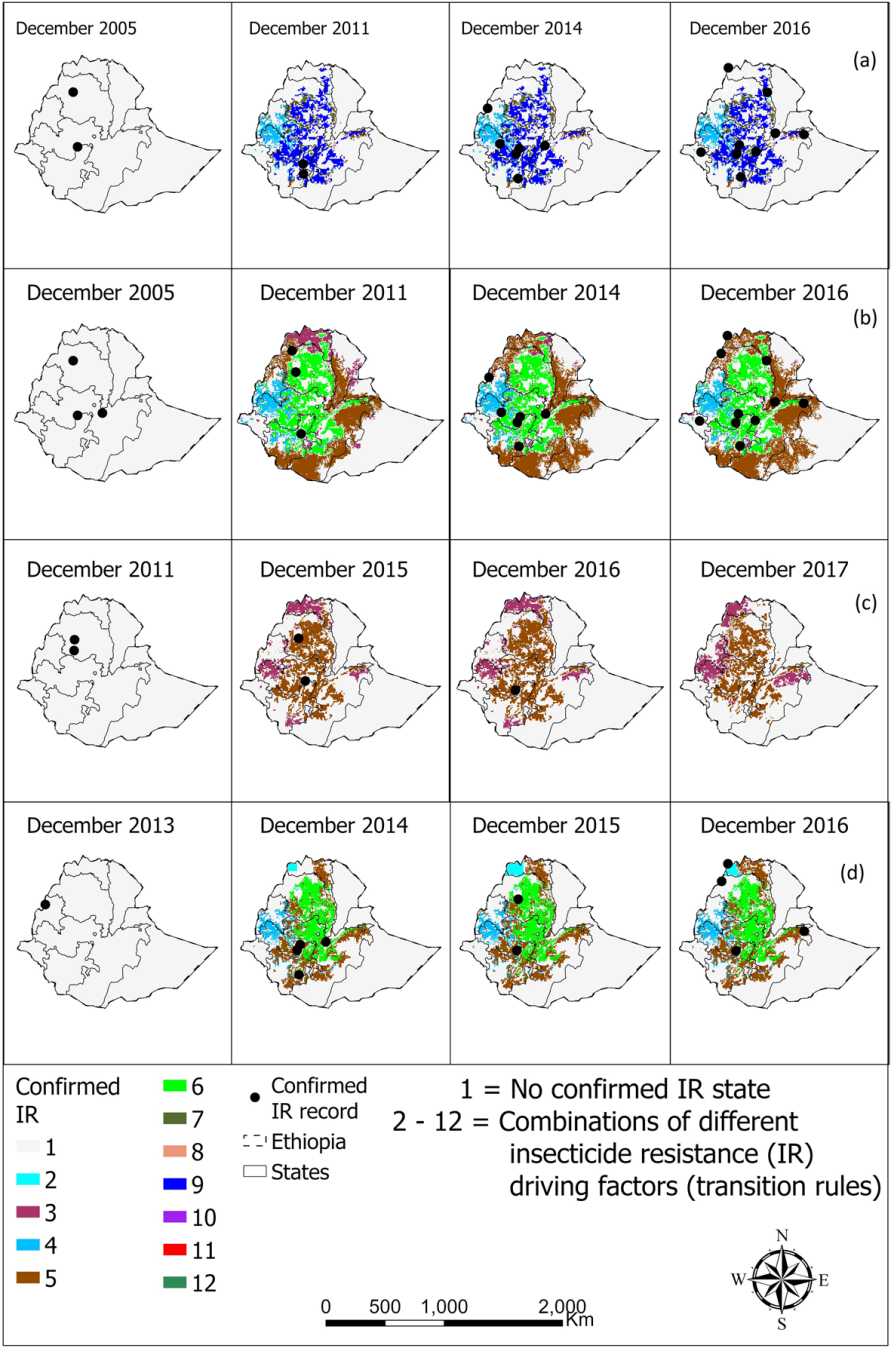
Spatio-temporal distribution of confirmed resistance in *Anopheles arabiensis* to **a** pyrethroid, **b** organochlorine, **c** carbamate, and **d** organophosphate in Ethiopia

**Fig. 8:**
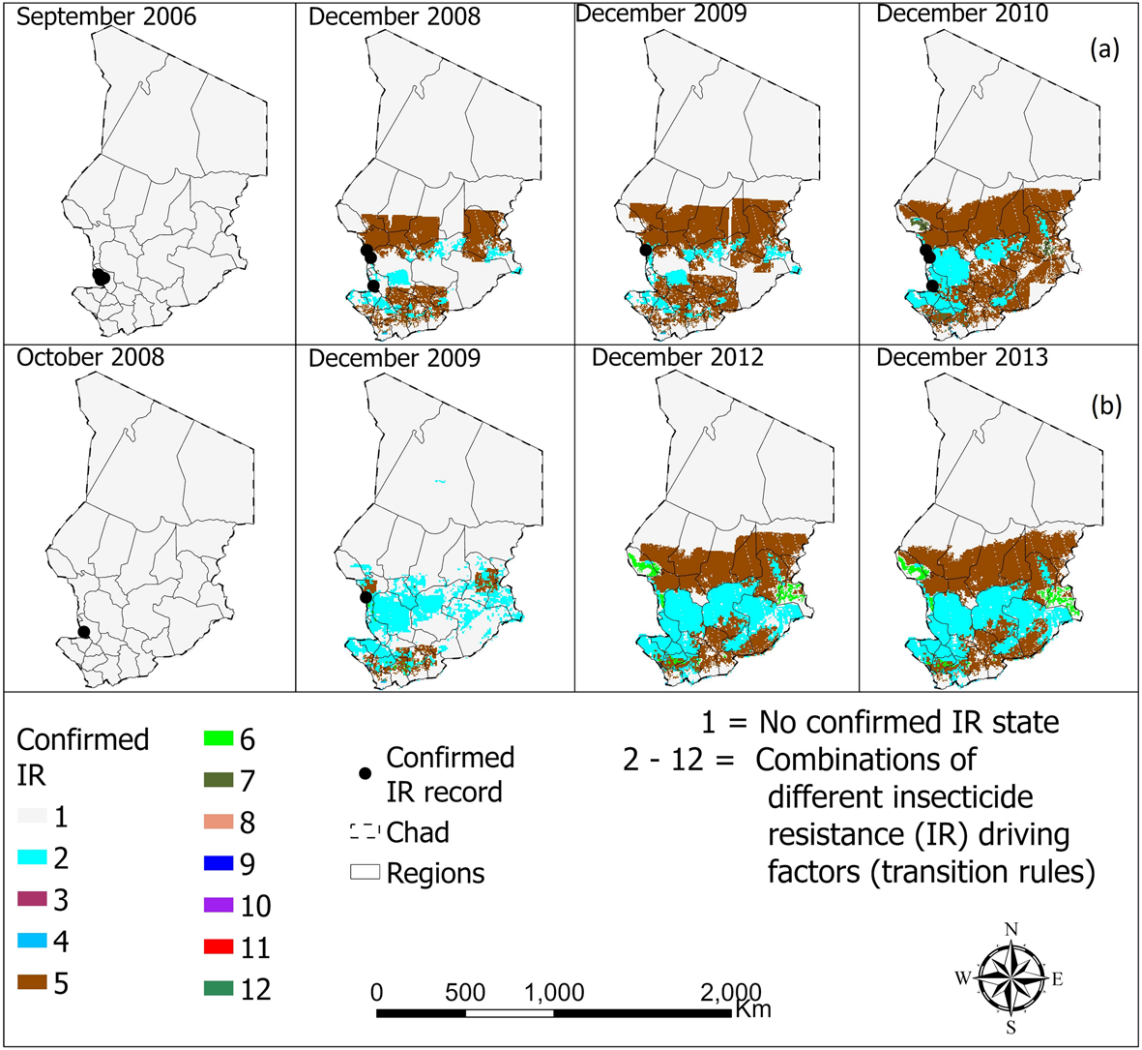
Spatio-temporal distribution of confirmed resistance in *Anopheles arabiensis* to **a** pyrethroid and **b** organochlorine in Chad

The CA models accurately represented the spatio-temporal distribution of confirmed IR in *An. arabiensis* in Ethiopia and Chad, achieving accuracies of over 80% in most cases following fine-tuning (Table 3). Furthermore, a significant portion of the georeferenced points indicating confirmed IR state coincided with the locations identified by the CA models as likely to contain confirmed IR state, as illustrated in Figs. 7 and 8. Table 3 details the accuracy levels achieved by comparing the actual versus predicted spatio-temporal distribution of confirmed insecticide resistance (IR) in *An. arabiensis* across various countries throughout the entire study duration. Conversely, Figs. 7 to 8 offer a visual depiction of the confirmed IR distribution for specific, selected years within the study period, highlighting temporal variations and trends.

**Table 3:** Accuracy scores obtained following the validation of our cellular automata models’ outputs for confirmed insecticide resistance IR state in *Anopheles arabiensis* in Ethiopia and Chad

	***Anopheles arabiensis***							
	**Ethiopia**				**Chad**			
**Year**	**Pyrethroid**	**Organochlorine**	**Carbamate**	**Organophosphate**	**Pyrethroid**	**Organochlorine**	**Carbamate**	**Organophosphate**
2001	-	-	-	-	-	-	-	-
2002	-	-	-	-	-	-	-	-
2003	-	-	-	-	-	-	-	-
2004	-	-	-	-	-	-	-	-
2005	-	-	-	-	-	-	-	-
2006	-	-	-	-	-	-	-	-
2007	-	-	-	-	-	-	-	-
2008	-	-	-	-	1.0000	-	-	-
2009	-	-	-	-	1.0000	1.0000	-	-
2010	-	-	-	-	1.0000	-	-	-
2011	1.0000	1.0000	-	-	-	-	-	-
2012	-	-	-	-	-	-	-	-
2013	1.0000	1.0000	-	-	-	-	-	-
2014	1.0000	0.8333	-	0.7500	-	-	-	-
2015	1.0000	0.8462	1.0000	0.5000	-	-	-	-
2016	0.7778	0.7778	1.0000	1.0000	-	-	-	-
2017	-	-	-	-	-	-	-	-
**Mean**	**0.9556**	**0.8839**	**1.0000**	**0.7500**	**1.0000**	**1.0000**	**-**	**-**

*CA* cellular automata

### Cellular automata (CA) model validation

**Fig. 9:**
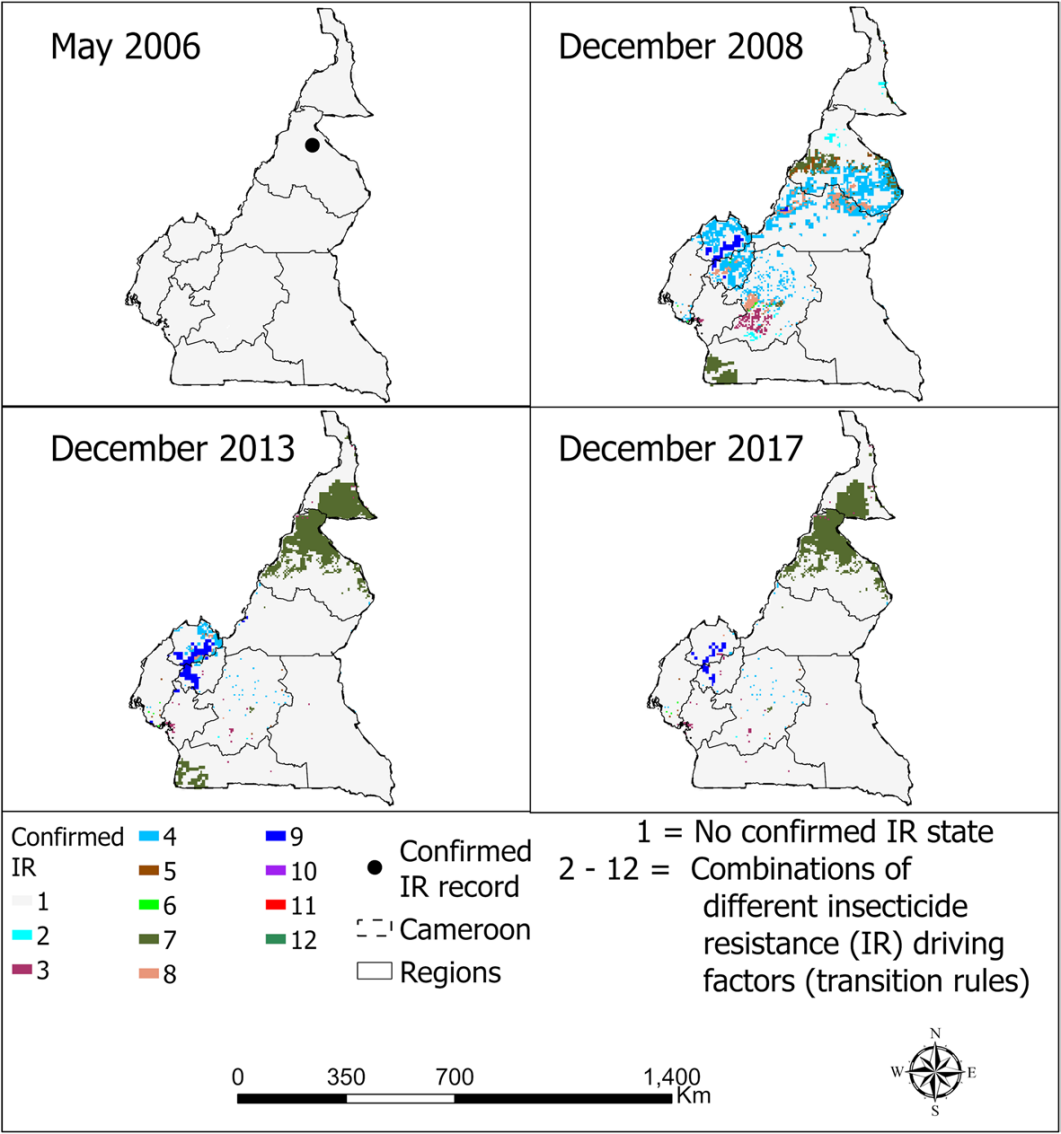
Spatio-temporal distribution of confirmed resistance in *Anopheles arabiensis* to pyrethroids in Cameroon for selected year

Due to limited data points for confirmed IR in *An. arabiensis* for many countries, with some having 1 or 2 records for the entire period considered, validation of the models was challenging. To address this, the CA models were deployed to Cameroon with acceptable data points, and the results compared with those from previous studies. This comparison revealed that CA model outputs as shown in Fig. 9, coincided with locations where previous studies reported IR. The maps also coincide with regions of occurrence of *An. arabiensis* consistency. This clear demonstration underscores the strength of our methodology, which possesses the capability to predict patterns and occurrences in regions where the model did not have access to data during the calibration exercise. In essence, it showcases the robustness and generalizability of our model beyond the specific data points used for its development. This ability to extrapolate and generate accurate predictions in data-scarce areas is a significant advantage of our approach and contributes to its effectiveness in understanding and characterizing the spatial and temporal dynamics of confirmed insecticide resistance in malaria vectors.

## Discussion

The developed CA models exhibit a remarkable capability to effectively capture the spatio-temporal dynamics of confirmed insecticide IR state in *An. gambiae* complex and *An. arabiensis* populations. This efficacy is clearly demonstrated by the strong agreement between the actual confirmed IR incidences and the CA outputs generated by the CA models. Moreover, the high accuracy in scores obtained during out-of-sample validation highlight the reliability and precision of our modelling approach, which has also been demonstrated in literature [22]. The models' ability to accurately depict the distribution of confirmed IR can be attributed to; firstly, the inclusion of transition rules that account for the variability of IR in different agro-ecological zones contributes to their effectiveness. This consideration ensures that the models can adapt to the specific conditions and factors influencing IR within each zone, resulting in a more accurate representation of the spatio-temporal dynamics.

Secondly, a significant enhancement to the methodology is the incorporation of an additional term in the CA’s function, allowing for the modeling of the spatio-temporal distribution of confirmed IR states. This extension enables the models to account for the possibility of confirmed IR emerging in areas that are not part of the immediate neighborhood cells. This is particularly valuable in situations where the spread of IR is not solely determined by neighboring cells but is influenced by the conditions prevailing in specific locations. Thus, the results emphasize that confirmed IR states are highly dependent on the unique conditions of locations, rather than solely on the spread from an initial point. The key driving factors identified through correlation analysis, chi-square tests, and existing literature include insecticide use, agricultural activities, human population density/counts, and environmental factors (temperature, humidity, and precipitation) [15, 17]. Notably, urban and built-up lands, croplands, and cropland/natural vegetation mosaics consistently exhibited higher proportions of confirmed IR compared to other LULC classes. This association can be explained by the increased presence of human populations, intensified agricultural activities, and higher insecticide usage in these areas, all of which contribute to a greater likelihood of confirmed IR. Previous studies have also highlighted the role of urbanization and various agricultural practices, such as rice, cotton, sugarcane, and vegetable farming, in promoting the emergence of IR within malaria vector populations in different regions. These findings underscore the complex interplay of environmental and human-related factors in shaping the dynamics of IR in mosquito vectors, emphasizing the need for region-specific approaches to IR management and control [16, 23–26].

Our findings also indicate a prevalent resistance among vector to organophosphates in barren land. These areas are often characterized by irrigation practices to facilitate agriculture, as noted in [27]. The agriculture activities in such drylands are susceptible to severe pest infestations, resulting in high use of inorganic pesticides, including organophosphates, to combat these pests [27]. Additionally, the use of organophosphates extends beyond agriculture for the control of a wide range of public health pests across different land use and land cover (LULC) classes [28], Confirmed resistance of malaria vectors to organophosphates in irrigated regions has also been documented [29], suggesting that the extensive application of these chemicals is a likely catalyst for the development of resistance in both targeted and non-targeted insect populations, including vectors. Moreover, the widespread resistance of vectors to pyrethroids and organochlorines has led to an increased reliance on organophosphates and carbamates as alternative control measures. This shift raises concerns about the potential for vectors to develop resistance to these insecticides as well [30], highlighting a cycle of resistance development that could undermine efforts to manage vector populations effectively.

Furthermore, the significant correlation between environmental factors and IR in malaria vectors can be attributed to the ectothermic nature of these vectors. Ectothermic organisms, like malaria vectors, rely heavily on local environmental conditions for their survival and metabolic activities. Hence, it becomes crucial to consider environmental variables when monitoring IR, as they directly impact the vector populations [31]. Temperature plays a vital role in shaping the dynamics of IR in mosquito vectors. Temperature has a significant association with IR because it can regulate enzyme activities and gene expression, thereby influencing the metabolism of insecticides within the malaria vectors [32]. In harsh environmental conditions, the expression of enzymes in insects tends to increase, potentially leading to higher levels of resistance [32]. Previous studies have also demonstrated that mosquito vectors exposed to insecticides tend to have higher levels of enzyme expression [27, 29]. Therefore, temperature can significantly affect the efficacy of insecticides against African malaria vectors [33].

The observed significant association between insecticide usage and confirmed IR across various areas further highlights the role of insecticide pressure in driving IR in mosquito vectors. The intensive use of insecticides for malaria vector control, whether through ITNs or IRS, is a major contributing factor to the development of IR in mosquito populations [34]. The continued application of insecticides exerts selective pressure on the vector populations, favoring the survival and reproduction of individuals with resistance traits. As a result, IR becomes more prevalent within the mosquito vector populations over time. These findings underscore the importance of sustainable and strategic insecticide use in malaria control programs to mitigate the development of IR and ensure the continued effectiveness of vector control interventions.

While our model incorporated various factors associated with IR occurrence, there were factors, such as oil spillage, specific agricultural insecticide usage, and their concentration levels, that were not included due to data unavailability [35]. Because such IR driving factors vary in space and time, we believe that their inclusion could have further enhanced the robustness of the CA model in predicting IR dynamics. These additional variables, if considered, might have provided more comprehensive insights into the mechanisms driving IR in malaria vectors.

It is important to note that the data available for IR in the earlier years may not be as accurate and detailed as contemporary data, primarily due to differences in data collection methodologies, reporting, and geo-referencing. This potentially leads to discrepancy in data quality. However, in the recent years, advances in technology and improved surveillance systems, and standardization procedures of conducting susceptibility tests have led to more accurate and precise data. This discrepancy in data quality could partially explain instances where the model achieved lower accuracy scores.

Additionally, some of the countries used for model validation had limited initial and subsequent IR data records, making it challenging to conduct a comprehensive comparison between the actual and predicted maps. To overcome this limitation, we compared our model outputs with those of other studies. For instance, in Cameroon, our model's identification of areas with confirmed resistance in *An. arabiensis* coincided with habitat suitability maps for the same species from previous studies [36]. Generally, in addressing the challenge of confirmed IR prevalence, a significant obstacle is the absence of data on reported cases in many regions [37]. This gap necessitates the prediction of IR in areas lacking direct evidence. A case in point is the upper regions of Cameroon, where records of confirmed IR to *An. Arabiensis* are notably sparse. Intriguingly, this area is adjacent to the lower regions of Chad, which possess numerous records of confirmed IR, suggesting a consistency in the prediction of IR within the same agro-ecological zone. Such results further highlights lack of records of confirmed IR in some regions, possibly because no IR related study has been carried out there, or else, the findings have not been availed publicly. Hence, the results primarily highlight the scarcity of reported IR cases rather than inaccuracies in prediction leading to false positives. This scenario underscores the critical role of modeling in identifying potential hotspots of confirmed IR cases in regions yet to be directly investigated, thereby offering valuable insights into the spread and prevalence of IR where empirical data is limited. This external validation helped confirm the validity of our model predictions.

Another potential limitation is that some of the driver data were available only on a yearly or irregular basis, while the model was implemented on a monthly time step. This discrepancy in temporal resolution could have influenced the results, hence further research is needed to assess the impact of data temporality on the accuracy of the CA model. While our CA model provides valuable insights into the spatio-temporal dynamics of confirmed IR in malaria vectors, it is important to acknowledge these limitations and consider them when interpreting the results. Future research should aim to improve data availability and accuracy and explore additional factors that could contribute to IR in mosquito populations.

## Conclusions

The Cellular Automata (CA) model provides a robust characterization of phenotypic resistance in malaria vector populations across both space and time. It effectively accounts for variations in agro-ecological conditions and allows for the possibility of confirmed insecticide resistance (IR) occurring in locations that may not be in the immediate neighborhood of previously confirmed IR cases. This modeling approach is particularly valuable for identifying areas with the potential for malaria vector populations to develop resistance, thereby informing more effective management strategies for IR. One of the key strengths of this model is its adaptability to settings where limited susceptibility test data is available. By harnessing the power of CA, it becomes possible to gain insights into IR dynamics in regions with sparse or incomplete data, aiding in the early detection and mitigation of resistance. Furthermore, the CA models developed in this study can be applied in various contexts beyond malaria vector resistance modeling. They can be used in situations where a particular phenomenon is dependent on the unique conditions of a location, rather than solely relying on neighboring cells for its spread. This versatility opens new avenues for modeling and understanding complex spatial and temporal processes. In summary, the CA model presented in this study offers a powerful and flexible tool for characterizing phenotypic resistance in malaria vectors, which can contribute to more informed decision-making and effective interventions in the fight against malaria and other vector-borne diseases.

## Methods

### The study site

The study was conducted in parts of the African continent that bear the largest burden of malaria risk and have reported cases of IR in malaria vectors [1]. The study focused on Nigeria, Burkina Faso, Ethiopia, Mali, Cameroon, Ghana, Tanzania, Côte d’Ivoire, and Mozambique due to the consistent availability and the reliability of data records on IR in malaria vector populations, spanning from 2005 to 2017.

### Data

In this study, we used spatio-temporal data on IR in malaria vectors from the Vector Atlas (VA) database. This dataset encompasses georeferenced records detailing IR levels in mosquito vector populations across various classes of insecticides, namely pyrethroid, organochlorine, carbamate, and organophosphate. The IR state at each location is quantified as the percentage of vectors that survive (or perish) when subjected to susceptibility tests. Our study primarily focused on regions with extensive and consistent data records spanning over 15 years in Nigeria, Burkina Faso, Ethiopia, Mali, Cameroon, Ghana, Tanzania, Côte d’Ivoire, and Mozambique. By correlating the coordinates of these spatio-temporal IR data with corresponding IR driver values, we obtained information from various databases (Additional file 6: Table S5).

The IR driver variables were chosen based on a thorough review of existing literature and prior research endeavors around the modeling and mapping of IR distribution. The drivers encompass a range of critical factors that are known to influence the prevalence and spatial distribution of IR in mosquito vector populations. These factors include:

1. Human population density/count: The density and count of human populations in specific regions play a pivotal role in shaping IR dynamics. Areas with high human density often experience more extensive insecticide usage and consequently have a greater potential for the development and spread of IR [31].
2. Insecticide usage for vector control: The frequency and intensity of insecticide application for vector control significantly impact the selection pressure on mosquito populations. Areas with widespread insecticide use are more likely to witness the emergence and propagation of IR [23].
3. Agricultural activities: Agricultural practices can influence the presence of IR in malaria vectors. The use of insecticides in agriculture can contribute to the selection of resistance traits in mosquito populations [25].
4. Environmental/Climatic factors: Environmental and climatic conditions, such as temperature, humidity, and precipitation, can affect mosquito vector biology and behavior, which, in turn, can influence the development and spread of IR [5, 17].

A comprehensive summary of these IR drivers, along with their associated sources and relevance, is provided in Table 4. These drivers were carefully considered to construct a robust model that can effectively characterize the spatio-temporal dynamics of phenotypic resistance in malaria vector species.

**Table 4:** Potential drivers of insecticide resistance in malaria vectors

	**Human population**		**Agricultural activities**
1	Population count	23	Millet farming using all techniques
2	Population density	24	Millet irrigated portion
	**Insecticide use**	25	Cassava farming using all techniques
3	Insecticide-treated bed nets (ITN) use	26	Cassava irrigated portion
4	Indoor residual spraying (IRS); pyrethroid, organochlorine, carbamate, organophosphate, combined insecticides	27	Sorghum farming using all techniques
	**Agricultural activities**	28	Sorghum irrigated portion
5	Rice farming using all techniques	29	Wheat farming using all techniques
6	Rice irrigated portion	30	Wheat irrigated portion
7	Soybeans farming using all techniques	31	Vegetable farming
8	Soybeans irrigated portion	32	Vegetable irrigated
9	Groundnuts farming using all techniques	33	Banana plantains
10	Groundnuts irrigated portion	34	Yams
11	Other oil crops using all techniques		**Climatic/environmental drivers**
12	Other oil crops irrigated portion	35	Precipitation
13	Coffee farming using all techniques	36	Temperature minimum
14	Coffee irrigated portion	37	Temperature maximum
15	Potatoes farming using all techniques	38	Wind speed
16	Potatoes irrigated portion	39	Relative humidity
17	Sugarcane farming using all techniques	40	Solar radiation
18	Sugarcane irrigated portion	41	Surface water balance
19	Cotton farming using all techniques	42	Normalized difference vegetation index (NDVI)
20	Cotton irrigated portion	43	Enhanced vegetation index (EVI)
21	Maize farming using all techniques	44	Elevation
22	Maize irrigated portion		

### Data exploration

Prior to extracting values of IR drivers from various databases, we excluded non-georeferenced records to ensure data consistency and reliability. Subsequently, we conducted an in-depth exploratory data analysis (EDA) for each class of insecticide. The primary objective of this analysis was to identify the key drivers that significantly influenced occurrence of confirmed IR state in both space and time. The criteria for defining confirmed IR state were based on the guidelines provided by the WHO, where confirmed IR state is characterized by vector populations exhibiting a mortality rate of less than 90% within 60 min following susceptibility testing [38]. It is important to note that other states, such as possible resistance (mortality ranging from 90 to 97%) and susceptible resistance (mortality between 98 and100%), were also considered [38]. However, our specific focus was solely on confirmed resistance.

We further assessed the strength of the correlation between the identified IR drivers and the confirmed resistance state. Additionally, we explored the existence of any correlation or clustering patterns among the IR drivers themselves. To achieve this, we employed several EDA techniques tailored to the nature of the data. For continuous data, we utilized principal component analysis (PCA), correlation tests, and cluster analysis. On the other hand, for categorical data, we performed chi-square tests. One of the key metrics used to quantify the strength of correlation was the correlation coefficient (*r*), expressed mathematically as:

1 $$r = \frac{ \sum \left({x}_{i}-\overline{x }\right)\left({y}_{i}-\overline{y }\right)}{\sqrt{\sum {({x}_{i}-\overline{x })}^{2}\sum {({y}_{i}-\overline{y })}^{2}}}$$

where. $r$ = correlation coefficient ${x}_{i}$ and $\overline{x }$ are the values and mean of *x* variable respectively ${y}_{i}$ and $\overline{y }$ are the values and mean of *y* variable respectively.

The equation for chi-square statistic is.

2 $${\chi }^{2}= \sum_{i=1}^{k}\frac{{({O}_{i}-{E}_{i})}^{2}}{{E}_{i}}$$

where ${\chi }^{2}$ is the chi-square statistic ${O}_{i}$ is the observed frequency ${E}_{i}$ is the expected frequency

To identify the key drivers using PCA, our first step involved identifying the principal components (PCs) that collectively explained over 80% of the variation in the data based on cumulative proportion. Once we had identified these essential PCs, we then selected IR drivers that exhibited loading exceeding 0.25 from each of the identified PCs. We then used cluster analysis (dendrograms) to obtain clusters of the IR drivers. These clusters were instrumental in determining which drivers were closely related. The selection process for variables to retain or drop within these clusters was informed by various criteria. We considered the strength and significance of the correlation of each driver with the confirmed IR, as well as the correlations among the drivers within the same cluster. Additionally, we considered the loadings derived from the PCA outputs. In the process of determining which drivers to include, we also conducted a literature review to help us make informed decisions on which IR drivers from the clusters were most relevant and essential for our study. After obtaining the refined subset of IR drivers for use in our analysis, we implemented cellular automata (CA) models. We employed the CA approach, which allows for consideration of the potential initiation of the IR state at different locations, providing a more comprehensive understanding of IR dynamics. The CA models were specifically designed for *An. gambiae* complex and *An. arabiensis* and were implemented separately for each class of insecticide under consideration.

### Cellular automata (CA) approach

Cellular automata (CA) belongs to a group of spatially explicit models (SEM) which are constructed upon transition rules defining the progression of a geographical phenomenon [39]. SEM are computational methods rooted in geographical locations, allowing for replication of the dynamics of geographical phenomenon [40]. CA operates by utilizing a grid consisting of individuals cells each assigned a finite state. Over time, these cells undergo state changes governed by specified transition rules influenced by the state of neighboring cells. In the CA approach, the spatial domain is divided into grid cells, each initially assigned a state denoted as ${C}_{ij}^{t=0}$. The state of cell (i,j) at *t* + ∆*t* is expressed as;

3 $${C}_{ij}^{t+\Delta t}=f({C}_{ij}^{t},{O}_{ij}^{t},R)$$

where ${C}_{ij}^{t}$ is the cell state at time *t*, ${O}_{ij}^{t}$ is the state of cells in its neighborhood, *R* represents the transition rules, and ∆*t* is the time step.

In our study, we extended the CA model to encompass the potential for the state of interest, which is confirmed resistance, to emerge in cells that do not belong to the immediate neighborhood cells at time *t* + ∆*t*. This confirmed IR state could initiate in any location beyond the neighboring cells, if the prevailing conditions in that specific location permit the onset of the confirmed resistance state at a given time *t*. This extension allows for accounting for the possibility that “confirmed resistance” may develop in areas that are not in direct proximity to the current location. It acknowledges that the spatial distribution of IR is influenced not only by neighboring cells but also by conditions and factors that may impact IR emergence in more distant locations within the grid. This enhanced model provides a more comprehensive understanding of how “confirmed resistance” spreads and evolves in space and time. We represented this novel concept as.

4 $${C}_{ij}^{t+\Delta t}=\left\{\begin{array}{c}f({C}_{ij}^{t},{O}_{ij}^{t},R), {N}_{ij}^{t}=0\\ f({C}_{ij}^{t},{O}_{ij}^{t},R, {N}_{ij}^{t}), {N}_{ij}^{t}>0\end{array}\right.$$

where ${C}_{ij}^{t}$ is the cell state at time *t*, ${O}_{ij}^{t}$ is the state of cells in its neighborhood, R represents the transition rules, and ∆*t* is the time-step ${N}_{ij}^{t}$ is the cell which is not part of neighborhood cells and whose conditions at time *t* allows for the commencement of the state of interest, in this case, confirmed resistance to insecticide.

In the model, the appearance or disappearance of a confirmed IR state within a specific cell at a given time (*t*) depends on the current state of the IR drivers associated to that cell at time *t*. For instance, should temperature and other relevant IR drivers in the cell favor the prevalence of confirmed IR state to prevail, then it persists over time. However, if the temperature rises above a threshold where the vector with confirmed IR state cannot survive, applying the transition rules lead to the disappearance of confirmed IR state in that cell, at a given time. This *t* can be expressed mathematically as in Eq. 5;

5 $${C}_{ij}^{t+\Delta t}=\left\{\begin{array}{c} 1 \,if\, R\, or\, {N}_{ij}^{t}=True\\ 0\, otherwise\end{array}\right.$$

where ${C}_{ij}^{t+\Delta t}$ is the cell state at time *t* + ∆*t*. Here, a value of 1 signifies a cell in a confirmed IR state, indicating that the cell will maintain this state if the IR driver conditions are conducive to the persistence of confirmed IR within the mosquito population. Should these conditions deteriorate over time, cells previously in a confirmed IR state will lose this designation. Such changes in the IR driver conditions drive the dynamics of confirmed IR states. Similarly, this framework applies to the genesis of confirmed IR states in cells beyond the immediate vicinity. A cell transitions to a confirmed IR state if the IR driver conditions are favorable for the emergence of a mosquito population exhibiting confirmed IR.

### Model implementation

From the subset of IR drivers obtained after conducting EDA, we formulated the transition rules for the CA model, with a focus on each species and insecticide class. These transition rules governed the interaction of different IR drivers within cells of interest, that is neighborhood cells and cells within the study area, to determine whether the state of IR in such cell transitioned to confirmed IR or not. This underscored the importance of considering the values of the IR drivers at each cell at time step to ascertain the expected dynamics of confirmed IR state.

To determine these transition rules and set appropriate thresholds and association between IR drivers and confirmed IR state, we employed various analytical techniques including descriptive statistics, cross-tabulations, boxplots, proportion tables, pivot tables, and charts. Through summary statistics and literature review, we established boundaries for the continuous factors driving IR. This process involved setting the observed minimum and maximum values of these IR drivers as the lower and upper thresholds, respectively. For categorical variables, thresholds were determined by analyzing how their categories correlated with the confirmed IR state, utilizing proportionate analyses, pivot tables, and charts for this purpose. To refine our dataset further, we employed boxplots to identify and subsequently exclude potential outliers. In our quest to discern the conditions conducive to the emergence of confirmed IR states in cells removed from their immediate neighborhood, we undertook exploratory data analysis. This step was crucial for observing trends in confirmed IR states across varying combinations of IR drivers, with cross-tabulations and pivot tables being instrumental in this endeavor. Additionally, we pinpointed specific IR drivers that, even in isolation, were linked to higher frequencies of confirmed IR prevalence. The significance of these IR drivers was ascertained through correlation tests, aiding in the identification of unique driver combinations that consistently foster confirmed IR states in mosquito populations. Literature reviews further reinforced our findings, confirming that certain IR driver combinations indeed promote confirmed IR states. For instance, we found that mosquito populations from areas practicing rice farming through irrigation consistently exhibited confirmed IR states, regardless of other agricultural methods employed. Other significant combinations identified include areas engaging in both irrigated rice and vegetable farming with temperatures spanning 15 to 38 °C; and locales with insecticide coverage (ITN use and IRS) surpassing 0.76, alongside vegetable farming and similar temperature ranges, as supported by literature [41, 42]. These exploratory analyses underscore the concept that IR states can propagate from their point of origin to neighboring areas, a phenomenon supported by various studies [30, 43–45] and indicative of a diffusion process. Furthermore, the transition rules derived from our analyses are detailed in the code, available in the section dedicated to data and material availability.

Once the transition rules were established, we divided several countries into grid cells measuring 5 by 5 km. We then extracted the values of the IR driving factors from various databases, utilizing the centroids of each cell for the period spanning from 2000 to 2018. These countries included Ethiopia, Cameroon, Burkina Faso, Uganda, and Nigeria. The data were available on various temporal scales, including monthly, yearly, or at intervals of several years. To standardize the temporal aspect, we converted the data to a yearly basis and interpolated them to create monthly time steps, facilitating the use of monthly intervals during the model implementation.

**Fig. 10:**
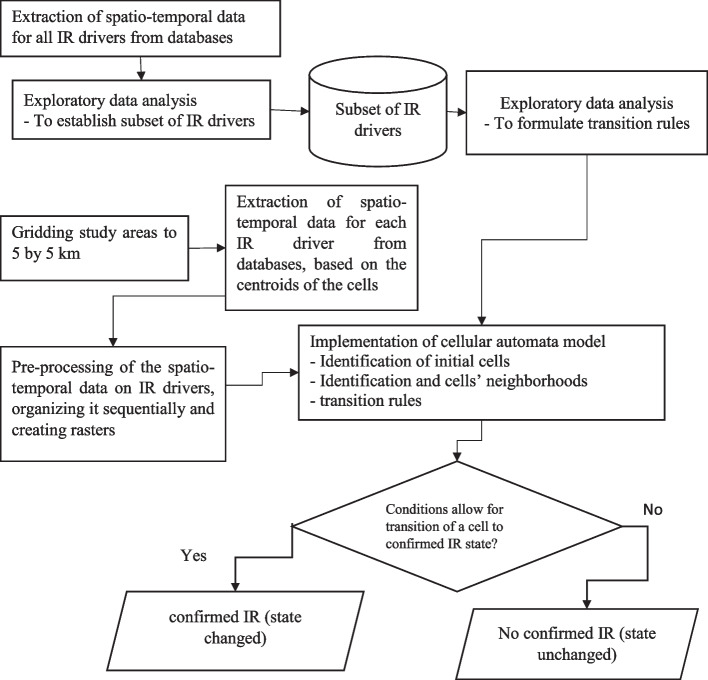
Summary flowchart of insect resistance model implementation

With the data of all IR drivers for each country for the entire period, we arranged them sequentially then converted the data to rasters. The resulting rasters were then stacked together, with centroids identifying each cell uniquely, while time identified each time step uniquely. Therefore, with transition rules formulated, and study areas gridded, and rasters for IR variables arranged sequentially, the next task was to identify the cell which contained the initial records of confirmed IR state for respective cell, determine its neighborhoods, then use the transition rules to establish whether the neighboring cells or those within the study area but not part of the neighborhoods cell would result into confirmed IR state or not. Therefore, to implement the CA models, we designated certain initial cells as points for the commencement of IR, assigning a value of 1 to cells with a confirmed IR state and 0 to those without. Subsequently, we executed the CA model based on the formulated rules, utilizing the Moore neighborhood configuration. Figure 10 illustrates the implementation of the model.

We implemented the CA model for both *An. gambiae* complex and *An*. *arabiensis* species, encompassing each of the four insecticide classes. The CA models were executed using R version 4.0.5 [46].

### Model assessment

Model fine-tuning emerged as a critical phase in our study, aiming to ensure the model’s capability to accurately reflect the dynamics of IR particularly within the *An. gambiae* complex and *An. arabiensis* found in several parts of Africa. For *An. gambiae* complex, we concentrated our fine-tuning efforts on Ethiopia, Cameroon, and Burkina Faso selected for their representation of Africa’s diverse agro-ecological landscapes. These countries served as the backdrop for adjusting our CA models to predict confirmed IR states for different classes of insecticides and vector species accurately. The fine-tuning process was thorough, with a primary focus on refining transition rules and thresholds, key determinants of the CA models’ ability to mimic the actual IR dynamics accurately. This refinement began with establishing transition rules based on identified thresholds, which are the ranges of predictor variables linked to confirmed IR states. Following this, the models were implemented, and their outputs were juxtaposed with actual IR case data to evaluate their accuracy. This evaluation informed further adjustments to the transition rules and thresholds, alongside the introduction of new rules catering to interactions among subsets of predictors, leading to iterative model implementations until the models proficiently mirrored the IR dynamics.

Subsequent to the fine-tuning phase, we proceeded to validate the models for confirmed IR within *An. gambiae* complex and *An. arabiensis*. Validation was executed using countries not previously involved in the EDA, rule formulation, or fine-tuning stages, such as Nigeria and Uganda for *An. gambiae* complex, and Cameroon for *An. arabiensis.* This approach, encompassing both spatial and temporal validation methods, aimed to assess if the models' predictions of IR locations aligned with actual reported IR incidences over time, thus ensuring the validation was conducted on an out-of-sample basis. Performance metrics, including the classification accuracy score, were employed to gauge the CA models’ effectiveness. This meticulous process of model fine-tuning and validation underscores our commitment to creating robust and accurate models capable of contributing significantly to the understanding and management of IR dynamics in mosquito populations across diverse African ecosystems.

6 $$Accuracy\, score= \frac{{T}_{p}+{T}_{N}}{{T}_{p}+{T}_{N}+{F}_{p}+{F}_{N}}$$

where; ${T}_{p}$ is the true positives ${T}_{p}$ is the true negatives ${F}_{p}$ is the false positives ${F}_{p}$ is the false negatives

In addition, we conducted a comparative analysis of the outputs of our model with existing models on mapping phenotypic resistance in Africa.

### Supplementary Information

Additional file 1. Table S1. Summary of clustering based on principal component analysis (PCA), correlation and cluster analysis using dendrogram

Additional file 2. Table S2. Correlation analysis

Additional file 3. Figures S1-S8. Figures S1-S8. FigS1- Variables clustering for pyrethroids insecticide class in Anopheles gambiae complex. FigS2 – Variables clustering for pyrethroids insecticide class in Anopheles gambiae complex FigS3 – Variables clustering for organochlorine insecticide class in Anopheles gambiae complex. FigS4 – Variables clustering for carbamate insecticide class in Anopheles gambiae complex. FigS5 – Variables clustering for organophosphate insecticide class in Anopheles arabiensis. FigS6 – Variables clustering for organochlorine insecticide class in Anopheles arabiensis. FigS7 – Variables clustering for carbamate insecticide class in Anopheles arabiensis. FigS8 – Variables clustering for organophosphate insecticide class in Anopheles arabiensis.

Additional file 4. Table S3. Chi square tests for associations between farming activities and confirmed insecticide resistance

Additional file 5. Table S4. Comparisons between cellular automata (CA) models’ outputs and actual confirmed IR state in An. gambiae complex 

Additional file 6. Table S5. Data sources [47–53].

## Data Availability

The datasets supporting the conclusions of this manuscript is available in the International Centre of Insect Physiology and Ecology Data Resources https://dmmg.icipe.org.

## Abbreviations

An.: Anopheles
CA: Cellular automata
DDT: Dichlorodiphenyltrichloroethane
EDA: Exploratory data analysis
EVI: Enhanced vegetation index
GAM: Generalized additive model
GIS: Geographic information system
GLM: Generalized linear model
IR: Insecticide resistance
IRS: Indoor residual spraying
ITN: Insecticide-treated bed nets
LULC: Land use and land cover
NDVI: Normalized difference vegetation index
PC: Principal component
PCA: Principal component analysis
VA: Vector atlas
WHO: World Health Organization
