# Supplementary material for: Spatio-temporal characterization of phenotypic resistance in malaria vector species
Source: BMC Biol. 2024 May 20;22:117. doi: 10.1186/s12915-024-01915-z (PMC11102860; doi:10.1186/s12915-024-01915-z)
Supplement: Supplementary file 1 — Additional file 1. Table S1. Summary of clustering based on principal component analysis (PCA), correlation and cluster analysis using dendrogram [file 12915_2024_1915_MOESM1_ESM.docx]

## Additional file 1: Table S1: Summary of clustering based on principal component analysis (PCA), correlation and cluster analysis using dendrogram

| *Anopheles gambiae* complex | | | |
| --- | --- | --- | --- |
| Pyrethroid | Correlation – significant variables | Dendrogram clusters | Subset of variables |
| **PC1**  Arabica coffee all tech  Arabica coffee irrigated portion  Banana all tech  Banana irrigated portion  Cotton all tech  Cotton irrigated portion  Maize all tech  Maize irrigated portion  Robusta coffee all tech  Robusta coffee irrigated portion  Sugarcane all tech  Sugarcane irrigated portion  Sweet potatoes all tech  Sweet potatoes irrigated portion  Wheat all tech  Wheat irrigated portion  Yams all tech  Yams irrigated portion  **PC2**  Precipitation  Temperature maximum  Relative humidity  Solar radiation  Surface water balance  Vapour pressure deficit  potential Evapotranspiration  Cloud area fraction  Climate moisture index  **PC3**  Temperature minimum  Solar radiation  Surface water balance  Cloud area fraction  Elevation  **PC4**  Population count  Population density  Rice all tech  PC5  Rice all tech  Rice irrigated portion  **PC6**  Wind speed  EVI  NDVI  groundnuts all tech  other oil crops all tech  soybeans all tech  **PC7**  EVI  NDVI  Groundnuts all tech  Soybeans all tech  **PC8**  Distance to water bodies  Groundnuts all tech  Groundnuts irrigated portion  Other oil crops irrigated portion | Temperature minimum  Relative humidity  Solar radiation  Potential evapotranspiration  EVI  Population count  Population density  Groundnuts all tech  Groundnuts irrigated portion  Other oil crops all tech  Other oil crops irrigated portion  Rice all tech  Arabica coffee all tech  Arabica coffee irrigated portion  Banana all tech  Banana irrigated portion  Cotton all tech  Cotton irrigated portion  Maize all tech  Maize irrigated portion  Robusta coffee all tech  Robusta coffee irrigated portion  Sugarcane all tech  Sugarcane irrigated portion  Sweet potatoes all tech  Sweet potatoes irrigated portion  Vegetables all tech  Vegetables irrigated portion  Wheat all tech  Wheat irrigated portion  Yams all tech  Yams irrigated portion | **Clusters A**  Vegetables all tech  Vegetables irrigated  **Clusters B**  Maize all tech  Maize irrigated portion  Banana all tech  Yams all tech  Sugarcane all tech  Sugarcane irrigated portion  Cotton all tech  Arabica coffee all tech  Sweet potatoes all tech  Wheat all tech  Robusta coffee all tech  Wheat irrigated portion  Cotton irrigated portion  Arabica coffee irrigated portion  Banana irrigated portion  Sweet potatoes irrigated portion  Robusta coffee irrigated portion  Yams irrigated portion  **Clusters C**  IRS pyrethroids  Precipitation  Temperature minimum  Temperature maximum  Wind speed  Relative humidity  Solar radiation  Surface water balance  Vapour pressure deficit  Potential evapotranspiration  Cloud area fraction  Climate moisture index  EVI  NDVI  Elevation  Distance to water bodies  Population count  Population density  Groundnuts all tech  Groundnuts irrigated portion  Other oil crops all tech  Other oil crops irrigated portion  Rice all tech  Rice irrigated portion  Soybeans all tech  Soybeans irrigated portion | Population density  Rice farming using all tech  Rice irrigated  Sugarcane all tech  Minimum temperature  Maximum temperature |
| Organochlorine | Correlation | Dendrogram clusters | Subset of variables |
| **PC1**  Arabica coffee all tech  Arabica coffee irrigated portion  Banana all tech  Banana irrigated portion  Cotton all tech  Cotton irrigated portion  Maize all tech  Maize irrigated portion  Robusta coffee all tech  Robusta coffee irrigated portion  Sugarcane all tech  Sugarcane irrigated portion  Sweet potatoes all tech  Sweet potatoes irrigated portion  Vegetables all tech  Vegetables irrigated portion  Wheat all tech  Wheat irrigated portion  Yams all tech  Yams irrigated portion  **PC2**  Precipitation  Temperature maximum  Relative humidity  Solar radiation  Surface water balance  Vapour pressure deficit  Potential evapotranspiration  Cloud area fraction  Climate moisture index  **PC3**  Precipitation  Temperature minimum  Solar radiation  Surface water balance  Cloud area fraction  Elevation  **PC4**  Population count  Population density  **PC5**  Rice all tech  Rice irrigated portion  **PC6**  EVI  NDVI  Other oil crops all tech  Soybeans all tech  organochlorine IRS  **PC7**  EVI  NDVI  Groundnuts irrigated portion  Other oil crops all tech  **PC8**  Distance to water bodies  Groundnuts all tech  Groundnuts irrigated portion  Other oil crops irrigated portion  Soybeans all tech | Precipitation  Temperature maximum  Wind speed  Relative humidity  Solar radiation  Surface water balance  Vapour pressure deficit  Potential evapotranspiration  Cloud area fraction  Climate moisture index  Population count  Population density  Groundnuts all tech  Groundnuts irrigated portion  organochlorine IRS | **Clusters A**  Vegetables all tech  Vegetables irrigated portion  **Clusters B**  Maize all tech  Banana all tech  Sugarcane all tech  Sugarcane irrigated portion  Yams all tech  Arabica coffee all tech  Sweet potatoes all tech  Cotton all tech  Wheat all tech  Wheat irrigated portion  Cotton irrigated portion  Arabica coffee irrigated portion  Banana irrigated portion  Robusta coffee irrigated portion  Sweet potatoes irrigated portion  Maize irrigated portion  Robusta coffee all tech  Yams irrigated portion  **Clusters C**  IRS organochlorine  Precipitation  Temperature minimum  Temperature maximum  Wind speed  Relative humidity  Solar radiation  Surface water balance  Vapour pressure deficit  Potential evapotranspiration  Cloud area fraction  Climate moisture index  EVI  NDVI  Elevation  Distance to water bodies  Population count  Population density  Groundnuts all tech  Groundnuts irrigated portion  Other oil crops all tech  Other oil crops irrigated portion  Rice all tech  Rice irrigated portion  Soybeans all tech  Soybeans irrigated portion | Population density  Rice farming using all tech  Rice irrigated  Minimum temperature  Maximum temperature |
| **Carbamate** | Correlation – significant variables | Dendrogram clusters | Subset of variables |
| **PC1**  Precipitation  Temperature maximum  Relative humidity  Surface water balance  Vapour pressure deficit  Potential evapotranspiration  Climate moisture index  **PC2**  Temperature minimum  Relative humidity  Solar radiation  Surface water balance  Cloud area fraction  Elevation  Wheat all tech  **PC3**  Population count  Population density  Robusta coffee irrigated portion  Yams irrigated portion  **PC4**  Arabica coffee all tech  Sugarcane all tech  Sugarcane irrigated portion  **PC5**  Population count  Population density  Groundnuts irrigated portion  Soybeans irrigated portion  Cotton irrigated portion  Wheat irrigated portion  **PC6**  Vegetables all tech  Vegetables irrigated portion  **PC7**  Rice all tech  Rice irrigated portion  **PC8**  Other oil crops all tech  Soybeans irrigated portion  Yams all tech  carbamate IRS  **PC9**  Soybeans irrigated portion  Cotton irrigated portion  Wheat all tech  Wheat irrigated portion  **PC10**  Other oil crops all tech  Rice all tech  Soybeans irrigated portion  Robusta coffee irrigated portion  Yams all tech  Yams irrigated portion  carbamate IRS  **PC11**  Other oil crops irrigated portion  Arabica coffee irrigated portion  Banana all tech  Banana irrigated portion  Sweet potatoes all tech  Sweet potatoes irrigated portion  **PC12**  EVI  NDVI  Distance to water bodies  Banana all tech  Cotton all tech  Sweet potatoes all tech  **PC13**  EVI  Distance to water bodies  Banana all tech  Sweet potatoes all tech  **PC14**  NDVI  Distance to water bodies  Other oil crops irrigated portion  Maize irrigated portion  Sweet potatoes irrigated portion  **PC15**  Other oil crops irrigated portion  Soybeans all tech  Maize all tech  Maize irrigated portion  Robusta coffee all tech  **PC16**  Cotton all tech  Maize all tech  Robusta coffee all tech  **PC17**  Other oil crops irrigated portion  Banana irrigated portion  Maize irrigated portion  Robusta coffee all tech  **PC18**  Groundnuts all tech  Other oil crops all tech  Other oil crops irrigated portion  Maize all tech  Sweet potatoes irrigated portion  **PC19**  Groundnuts all tech  Cotton all tech  Robusta coffee all tech  Sweet potatoes irrigated portion  Yams all tech | Precipitation  Temperature minimum  Relative humidity  Solar radiation  Vapour pressure deficit  potential Evapotranspiration  Cloud area fraction  Climate moisture index  ndvi  Elevation  Distance to water bodies  Population count  Population density  Groundnuts all tech  Soybeans all tech  Maize all tech  Robusta coffee all tech  Sugarcane all tech  Yams all tech | **Clusters A**  IRS carbamate  **Clusters B**  Vegetables all tech  Vegetables irrigated  **Clusters C**  Precipitation  Temperature minimum  Temperature maximum  Wind speed  Relative humidity  Solar radiation  Surface water balance  Vapour pressure deficit  Potential evapotranspiration  Cloud area fraction  Climate moisture index  EVI  ndvi  Elevation  Distance to water bodies  Population count  Population density  Groundnuts all tech  Groundnuts irrigated portion  Other oil crops all tech  Other oil crops irrigated portion  Rice all tech  Rice irrigated portion  Soybeans all tech  Soybeans irrigated portion  Arabica coffee all tech  Arabica coffee irrigated portion  Banana all tech  Banana irrigated portion  Cotton all tech  Cotton irrigated portion  Maize all tech  Maize irrigated portion  Robusta coffee all tech  Robusta coffee irrigated portion  Sugarcane all tech  Sugarcane irrigated portion  Sweet potatoes all tech  Sweet potatoes irrigated portion  portion  Wheat all tech  Wheat irrigated portion  Yams all tech  Yams irrigated portion  IRS carbamate | Population density  Rice farming using all tech  Rice irrigated  Sugarcane all tech  Minimum temperature  Maximum temperature |
| Organophosphates | Correlation – significant variables | Dendrogram clusters | Subset of variables |
| **PC1**  Arabica coffee all tech  Arabica coffee irrigated portion  Banana all tech  Banana irrigated portion  Cotton all tech  Cotton irrigated portion  Maize all tech  Maize irrigated portion  Robusta coffee all tech  Robusta coffee irrigated portion  Sugarcane all tech  Sugarcane irrigated portion  Sweet potatoes all tech  Sweet potatoes irrigated portion  Vegetables all tech  Vegetables irrigated portion  Wheat all tech  Wheat irrigated portion  Yams all tech  Yams irrigated portion  **PC2**  Precipitation  Temperature maximum  Relative humidity  Surface water balance  Vapour pressure deficit  Potential evapotranspiration  Climate moisture index  **PC3**  Precipitation  Temperature minimum  Relative humidity  Solar radiation  Surface water balance  Cloud area fraction  Elevation  **PC4**  Population count  Population density  **PC5**  Rice all tech  Rice irrigated portion  **PC6**  Other oil crops all tech  Soybeans all tech  organophospate IRS  **PC7**  EVI  NDVI  Groundnuts irrigated portion  **PC8**  EVI  Distance to water bodies  Groundnuts all tech  Groundnuts irrigated portion  Other oil crops irrigated portion  organophospate IRS | Wind speed  Potential evapotranspiration  EVI  Arabica coffee irrigated portion  Banana irrigated portion  Cotton all tech  Yams all tech | **Clusters A**  Vegetables all tech  Vegetables irrigated portion  **Clusters B**  Banana all tech  Maize all tech  Yams all tech  Arabica coffee all tech  Wheat all tech  Sweet potatoes all tech  Robusta coffee all tech  Wheat irrigated portion  Maize irrigated portion  Cotton irrigated portion  Arabica coffee irrigated portion  Banana irrigated portion  Sweet potatoes irrigated portion  Robusta coffee irrigated portion  Yams irrigated portion  Cotton all tech  Sugarcane all tech  Sugarcane irrigated portion  **Clusters C**  IRS organochlorine  Precipitation  Temperature minimum  Temperature maximum  Wind speed  Relative humidity  Solar radiation  Surface water balance  Vapour pressure deficit  Potential evapotranspiration  Cloud area fraction  Climate moisture index  EVI  NDVI  Elevation  Distance to water bodies  Population count  Population density  Groundnuts all tech  Groundnuts irrigated portion  Other oil crops all tech  Other oil crops irrigated portion  Rice all tech  Rice irrigated portion  Soybeans all tech  Soybeans irrigated portion | Population density  Rice farming using all tech  Rice irrigated  Minimum temperature  Maximum temperature |
| *Anopheles arabiensis* |  |  |  |
| Pyrethroid | Correlation | Dendrogram clusters | Subset of variables |
| **PC1**  Arabica coffee all tech  Arabica coffee irrigated portion  Banana all tech  Banana irrigated portion  Cotton all tech  Cotton irrigated portion  Maize all tech  Maize irrigated portion  Robusta coffee all tech  Robusta coffee irrigated portion  Sugarcane all tech  Sugarcane irrigated portion  Sweet potatoes all tech  Sweet potatoes irrigated portion  Vegetables all tech  Vegetables irrigated portion  Wheat all tech  Wheat irrigated portion  Yams all tech  Yams irrigated portion  **PC2**  Temperature minimum  Relative humidity  Solar radiation  Surface water balance  EVI  NDVI  Elevation  **PC3**  Temperature maximum  Surface water balance  Vapour pressure deficit  Potential evapotranspiration  Cloud area fraction  Climate moisture index  pyrethroid IRS  **PC4**  Wind speed  Population count  Population density  **PC5**  Groundnuts all tech  Rice all tech  Rice irrigated portion  Soybeans all tech  **PC6**  Precipitation  Solar radiation  potential Evapotranspiration  Climate moisture index  Rice irrigated portion | Temperature minimum  Temperature maximum  Surface water balance  Vapour pressure deficit  Elevation  Soybeans all tech | **Clusters A**  Maize all tech  Vegetables all tech  Banana all tech  Yams all tech  Cotton all tech  Sugarcane all tech  Sugarcane irrigated portion  Maize irrigated portion  Vegetables irrigated portion  Arabica coffee all tech  Wheat all tech  Wheat irrigated portion  Cotton irrigated portion  Banana irrigated portion  Yams irrigated portion  IRS pyrethroid  Sweet potatoes irrigated portion  Arabica coffee irrigated portion  Robusta coffee all tech  Robusta coffee irrigated portion  Sweet potatoes all tech  **Clusters B**  Precipitation  Temperature minimum  Temperature maximum  Wind speed  Relative humidity  Solar radiation  Surface water balance  Vapour pressure deficit  potential Evapotranspiration  Cloud area fraction  Climate moisture index  EVI  NDVI  Elevation  Distance to water bodies  Population count  Population density  Groundnuts all tech  Groundnuts irrigated portion  Other oil crops all tech  Other oil crops irrigated portion  Rice all tech  Rice irrigated portion  Soybeans all tech  Soybeans irrigated portion | Population density  Rice farming using all tech  Rice irrigated  Minimum temperature  Maximum temperature |
| Organochlorine | Correlation | Dendrogram clusters | Subset of variables |
| **PC1**  Arabica coffee all tech  Arabica coffee irrigated portion  Banana all tech  Banana irrigated portion  Cotton all tech  Cotton irrigated portion  Maize all tech  Maize irrigated portion  Robusta coffee all tech  Robusta coffee irrigated portion  Sugarcane all tech  Sugarcane irrigated portion  Sweet potatoes all tech  Sweet potatoes irrigated portion  Vegetables all tech  Vegetables irrigated portion  Wheat all tech  Wheat irrigated portion  Yams all tech  Yams irrigated portion  **PC2**  Precipitation  Temperature minimum  Temperature maximum  Surface water balance  Climate moisture index  NDVI  Elevation  Groundnuts all tech  **PC3**  Wind speed  Relative humidity  Solar radiation  Vapour pressure deficit  Potential evapotranspiration  Cloud area fraction  **PC4**  Population count  Population density  **PC5**  Cloud area fraction  Rice all tech  Rice irrigated portion | organochlorine  Precipitation  Temperature maximum  Wind speed  Relative humidity  Solar radiation  Surface water balance  Vapour pressure deficit  Potential evapotranspiration  Cloud area fraction  Climate moisture index  Population count  Population density  Groundnuts all tech  Groundnuts irrigated portion | **Clusters A**  Vegetables all tech  Maize all tech  Banana all tech  Maize irrigated portion  Vegetables irrigated portion  Cotton all tech  Sweet potatoes all tech  Robusta coffee all tech  Sugarcane all tech  Sugarcane irrigated portion  Arabica coffee all tech  Yams all tech  Wheat all tech  Wheat irrigated portion  Cotton irrigated portion  Banana irrigated portion  Arabica coffee irrigated portion  Robusta coffee irrigated portion  Sweet potatoes irrigated portion  Yams irrigated portion  **Clusters B**  Precipitation  Temperature minimum  Temperature maximum  Wind speed  Relative humidity  Solar radiation  Surface water balance  Vapour pressure deficit  Potential evapotranspiration  Cloud area fraction  Climate moisture index  EVI  NDVI  Elevation  Distance to water bodies  Population count  Population density  Groundnuts all tech  Groundnuts irrigated portion  Other oil crops all tech  Other oil crops irrigated portion  Rice all tech  Rice irrigated portion  Soybeans all tech  Soybeans irrigated portion  IRS organochlorine | Population density  Rice farming using all tech  Rice irrigated  Minimum temperature  Maximum temperature |
| **Carbamate** | Correlation | Dendrogram clusters | Subset of variables |
| **PC1**  Temperature minimum  Temperature maximum  Surface water balance  Vapour pressure deficit  Elevation  Sweet potatoes all tech  **PC2**  Precipitation  Climate moisture index  Maize irrigated portion  Sugarcane irrigated portion  Vegetables irrigated portion  **PC3**  Wind speed  Relative humidity  Cloud area fraction  Distance to water bodies  Soybeans all tech  **PC4**  Solar radiation  Potential evapotranspiration  EVI  NDVI  Carbamate IRS  **PC5**  Relative humidity  Cloud area fraction  Other oil crops all tech  Maize irrigated portion  Robusta coffee all tech  Sugarcane all tech  Sugarcane irrigated portion  Vegetables irrigated portion  **PC6**  Solar radiation  Potential evapotranspiration  Population count  Population density  Other oil crops all tech  Rice all tech  Sugarcane all tech  **PC7**  Arabica coffee all tech  Vegetables all tech  **PC8**  Rice all tech  Rice irrigated portion  **PC9**  Population count  Population density  Other oil crops irrigated portion  Soybeans all tech  Cotton all tech  carbamate IRS | Relative humidity  Maize all tech | **Clusters A**  Relative humidity  Cloud area fraction  **Clusters B**  Population count  Population density  **Clusters C**  Wind speed  Temperature minimum  Temperature maximum  Surface water balance  **Clusters D**  Vegetables all tech  Climate moisture index  Maize all tech  NDVI  Distance to water bodies  Groundnuts all tech  Groundnuts irrigated portion  Other oil crops all tech  Other oil crops irrigated portion  Rice all tech  Rice irrigated portion  Soybeans all tech  Soybeans irrigated portion  Arabica coffee all tech  Arabica coffee irrigated portion  Banana all tech  Banana irrigated portion  Cotton irrigated portion  Maize irrigated portion  Robusta coffee all tech  Robusta coffee irrigated portion  Sugarcane all tech  Sugarcane irrigated portion  Sweet potatoes all tech  Sweet potatoes irrigated portion  Vegetables irrigated portion  Wheat all tech  Wheat irrigated portion  Yams all tech  Yams irrigated portion  IRS carbamate  **Clusters E**  EVI  Elevation  **Clusters F**  Precipitation  Solar radiation  Vapour pressure deficit  Potential evapotranspiration | Population density  Rice farming using all tech  Rice irrigated |
| Organophosphates | Correlation | Dendrogram clusters | Subset of variables |
| **PC1**  Temperature minimum  Temperature maximum  Surface water balance  Elevation  Maize all tech  Sweet potatoes all tech  **PC2**  Wind speed  Distance to water bodies  Soybeans all tech  Cotton all tech  Vegetables all tech  **PC3**  Precipitation  Relative humidity  Cloud area fraction  Climate moisture index  Maize irrigated portion  Sugarcane irrigated portion  **PC4**  Groundnuts all tech  Rice all tech  Rice irrigated portion  Maize irrigated portion  Robusta coffee all tech  Sugarcane irrigated portion  Vegetables irrigated portion  **PC5**  Solar radiation  Potential evapotranspiration  **PC6**  EVI  NDVI  Other oil crops all tech  Banana all tech  Sugarcane all tech  **PC7**  Rice irrigated portion  Arabica coffee all tech  Robusta coffee all tech  Wheat all tech  PC8  Solar radiation  Population count  Population density  Yams all tech  organophosphate IRS | Maize all tech | **Clusters A**  Relative humidity  Cloud area fraction  **Clusters B**  Population count  Population density  **Clusters C**  Climate moisture index  Maize all tech  EVI  NDVI  Elevation  Distance to water bodies  Groundnuts all tech  Groundnuts irrigated portion  Other oil crops all tech  Other oil crops irrigated portion  Rice all tech  Rice irrigated portion  Soybeans all tech  Soybeans irrigated portion  Arabica coffee all tech  Arabica coffee irrigated portion  Banana all tech  Banana irrigated portion  Cotton all tech  Cotton irrigated portion  Maize irrigated portion  Robusta coffee all tech  Robusta coffee irrigated portion  Sugarcane all tech  Sugarcane irrigated portion  Sweet potatoes all tech  Sweet potatoes irrigated portion  Vegetables all tech  Vegetables irrigated portion  Wheat all tech  Wheat irrigated portion  Yams all tech  Yams irrigated portion  IRS organophosphate  **Clusters D**  Wind speed  Temperature minimum  Temperature maximum  Surface water balance  **Clusters E**  Precipitation  Solar radiation  Vapour pressure deficit  Potential evapotranspiration | Population density  Rice farming using all tech  Rice irrigated |

AG - *Anopheles gambiae* complex, AA – *Anopheles arabiensis*
