# Supplementary material for: Spatio-temporal characterization of phenotypic resistance in malaria vector species
Source: BMC Biol. 2024 May 20;22:117. doi: 10.1186/s12915-024-01915-z (PMC11102860; doi:10.1186/s12915-024-01915-z)
Supplement: Supplementary file 2 — Additional file 2. Table S2. Correlation analysis [file 12915_2024_1915_MOESM2_ESM.docx]

## Additional file 2: Table S2. Correlation analysis

|  | Confirmed resistance in *An. gambiae* complex | | | | Confirmed resistance in *An. arabiensis* | | | |
| --- | --- | --- | --- | --- | --- | --- | --- | --- |
|  | pyrethroid | organochlorine | carbamate | organophosphate | pyrethroid | organochlorine | carbamate | organophosphate |
| Percent mortality |  |  |  |  |  |  |  |  |
| Precipitation | -0.012 | -0.154^***^ | -0.129^**^ | 0.048 | -0.003 | -0.154^***^ | -0.454 | -0.732 |
| Minimum temperature | -0.038^**^ | -0.024 | -0.210^***^ | 0.129 | 0.189^*^ | -0.024 | -0.034 | 0.565 |
| Maximum temperature | -0.007 | 0.153^***^ | 0.079 | 0.125 | 0.425^***^ | 0.153^***^ | 0.309 | 0.78 |
| Wind speed | 0.02 | 0.152^***^ | -0.039 | 0.146^*^ | -0.129 | 0.152^***^ | 0.134 | 0.312 |
| Relative humidity | -0.039^**^ | -0.209^***^ | -0.214^***^ | -0.025 | 0.063 | -0.209^***^ | -0.572^*^ | -0.603 |
| Solar radiation | 0.067^***^ | 0.180^***^ | 0.226^***^ | 0.042 | -0.059 | 0.180^***^ | 0.406 | 0.672 |
| Surface water balance | -0.023 | 0.072^*^ | -0.043 | 0.143 | 0.288^***^ | 0.072^*^ | 0.03 | 0.64 |
| Vapour pressure deficit | 0.002 | 0.173^***^ | 0.127^**^ | 0.121 | 0.226^**^ | 0.173^***^ | 0.376 | 0.61 |
| Potential Evapotranspiration | 0.039^**^ | 0.175^***^ | 0.134^**^ | 0.174^*^ | 0.049 | 0.175^***^ | 0.332 | 0.623 |
| Cloud area fraction | -0.002 | -0.125^***^ | -0.142^**^ | -0.007 | 0.075 | -0.125^***^ | -0.315 | -0.74 |
| Climate moisture index | -0.019 | -0.172^***^ | -0.140^**^ | 0.007 | -0.011 | -0.172^***^ | -0.524 | -0.738 |
| EVI | -0.118^***^ | 0.002 | -0.061 | -0.190^**^ | -0.057 | 0.002 | -0.455 | 0.299 |
| NDVI | -0.019 | -0.005 | -0.177^***^ | 0.083 | 0.029 | -0.005 | -0.193 | 0.69 |
| Elevation | 0.022 | -0.031 | 0.239^***^ | -0.12 | -0.264^***^ | -0.031 | 0.236 | -0.841 |
| Distance to water bodies | 0.022 | 0.023 | 0.167^***^ | 0.044 | 0.142 | 0.023 | -0.016 | 0.776 |
| Population count | -0.123^***^ | -0.101^***^ | -0.146^**^ | -0.068 | -0.041 | -0.101^***^ | 0.288 | -0.237 |
| Population density | -0.123^***^ | -0.109^***^ | -0.141^**^ | -0.066 | -0.062 | -0.109^***^ | 0.284 | -0.236 |
| Groundnuts all tech | 0.073^***^ | 0.116^***^ | 0.109^*^ | 0.097 | 0.086 | 0.116^***^ | -0.358 | -0.813 |
| Groundnuts irrigated portion | 0.036^*^ | 0.104^***^ | 0.046 | 0.111 |  | 0.104^***^ |  |  |
| Other oil crops all tech | 0.095^***^ | 0.024 | 0.086 | -0.072 | -0.125 | 0.024 | 0.118 | -0.922 |
| Other oil crops irrigated portion | -0.043^**^ | -0.033 |  | -0.058 | 0.097 | -0.033 |  |  |
| Rice all tech | -0.064^***^ | 0.003 | 0.088 | 0.033 | -0.046 | 0.003 | -0.423 | -0.129 |
| Rice irrigated portion | -0.024 | 0.052 | 0.061 | 0.057 | -0.115 | 0.052 | -0.269 | -0.922 |
| Soybeans all tech | -0.012 | -0.036 | 0.151^**^ | -0.022 | 0.172^*^ | -0.036 | 0.269 | 0.628 |
| Soybeans irrigated portion | 0.005 | 0.024 |  |  |  | 0.024 |  |  |
| Arabica coffee all tech | -0.034^*^ | -0.041 | 0.063 | -0.029 | -0.096 | -0.041 |  |  |
| Arabica coffee irrigated portion | -0.034^*^ | -0.041 | 0.041 | -0.179^*^ | -0.096 | -0.041 |  |  |
| Banana all tech | -0.034^*^ | -0.041 | 0 | 0.021 | -0.096 | -0.041 | 0.333 | 0.839 |
| Banana irrigated portion | -0.034^*^ | -0.041 | 0.004 | -0.146^*^ | -0.096 | -0.041 |  |  |
| Cotton all tech | -0.034^*^ | -0.041 | 0.036 | 0.162^*^ | -0.096 | -0.041 | 0.049 | -0.083 |
| Cotton irrigated portion | -0.034^*^ | -0.041 | 0.029 | 0.101 | -0.096 | -0.041 |  |  |
| Caize all tech | -0.034^*^ | -0.041 | 0.127^**^ | -0.004 | -0.096 | -0.041 | -0.558^*^ | -0.976^*^ |
| Maize irrigated portion | -0.034^*^ | -0.041 | 0.047 | 0.133 | -0.096 | -0.041 | -0.28 | -0.922 |
| Robusta coffee all tech | -0.034^*^ | -0.041 | -0.280^***^ | 0.028 | -0.096 | -0.041 |  |  |
| Robusta coffee irrigated portion | -0.034^*^ | -0.041 | 0.083 | -0.007 | -0.096 | -0.041 |  |  |
| Sugarcane all tech | -0.034^*^ | -0.041 | 0.110^*^ | 0.008 | -0.096 | -0.041 | 0.077 | -0.922 |
| Sugarcane irrigated portion | -0.034^*^ | -0.041 | 0.081 | 0.009 | -0.096 | -0.041 | -0.238 | -0.922 |
| Sweet potatoes all tech | -0.034^*^ | -0.041 | -0.011 | -0.001 | -0.096 | -0.041 | 0.056 | -0.885 |
| Sweet potatoes irrigated portion | -0.034^*^ | -0.041 | 0.074 | 0.055 | -0.096 | -0.041 |  |  |
| Vegetables all tech | -0.038^**^ | -0.009 | -0.012 | -0.078 | -0.096 | -0.009 | -0.062 | 0.8 |
| Vegetables irrigated portion | -0.038^**^ | -0.009 | 0.045 | 0.076 | -0.096 | -0.009 | -0.159 | -0.922 |
| Wheat all tech | -0.034^*^ | -0.041 | 0.064 | 0.03 | -0.096 | -0.041 |  |  |
| Wheat irrigated portion | -0.034^*^ | -0.041 | 0.035 | 0.108 | -0.096 | -0.041 |  |  |
| Yams all tech | -0.034^*^ | -0.041 | -0.115^*^ | -0.166^*^ | -0.096 | -0.041 | 0.2 | 0.634 |
| Yams irrigated portion | -0.034^*^ | -0.041 | 0.083 | -0.007 | -0.096 | -0.041 |  |  |
| IRS | -0.01 | -0.087^**^ | 0.083 | 0.062 |  | 0.02 | 0.317 |  |
