# Supplementary material for: Spatio-temporal characterization of phenotypic resistance in malaria vector species
Source: BMC Biol. 2024 May 20;22:117. doi: 10.1186/s12915-024-01915-z (PMC11102860; doi:10.1186/s12915-024-01915-z)
Supplement: Supplementary file 3 — Additional file 3. Figures S1-S8. Figures S1-S8. FigS1- Variables clustering for pyrethroids insecticide class in Anopheles gambiae complex. FigS2 – Variables clustering for pyrethroids insecticide class in Anopheles gambiae complex FigS3 – Variables clustering for organochlorine insecticide class in Anopheles gambiae complex. FigS4 – Variables clustering for carbamate insecticide class in Anopheles gambiae complex. FigS5 – Variables clustering for organophosphate insecticide class in Anopheles arabiensis. FigS6 – Variables clustering for organochlorine insecticide class in Anopheles arabiensis. FigS7 – Variables clustering for carbamate insecticide class in Anopheles arabiensis. FigS8 – Variables clustering for organophosphate insecticide class in Anopheles arabiensis. [file 12915_2024_1915_MOESM3_ESM.docx]

## Additional file 3: Figures S1 – S8: Outputs of Dendrograms

Figure S1


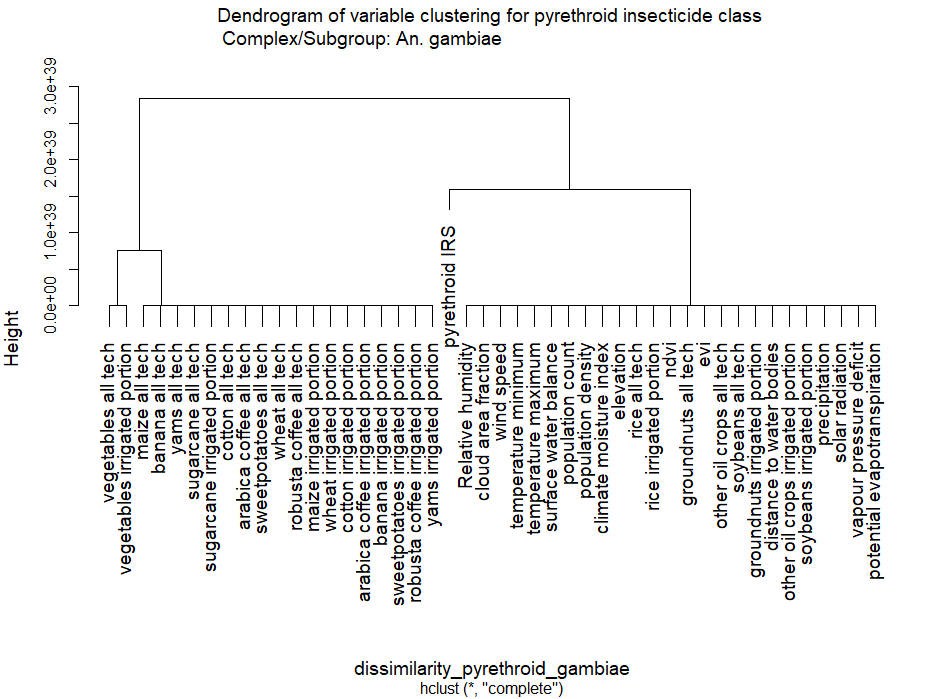


Figure S2

**
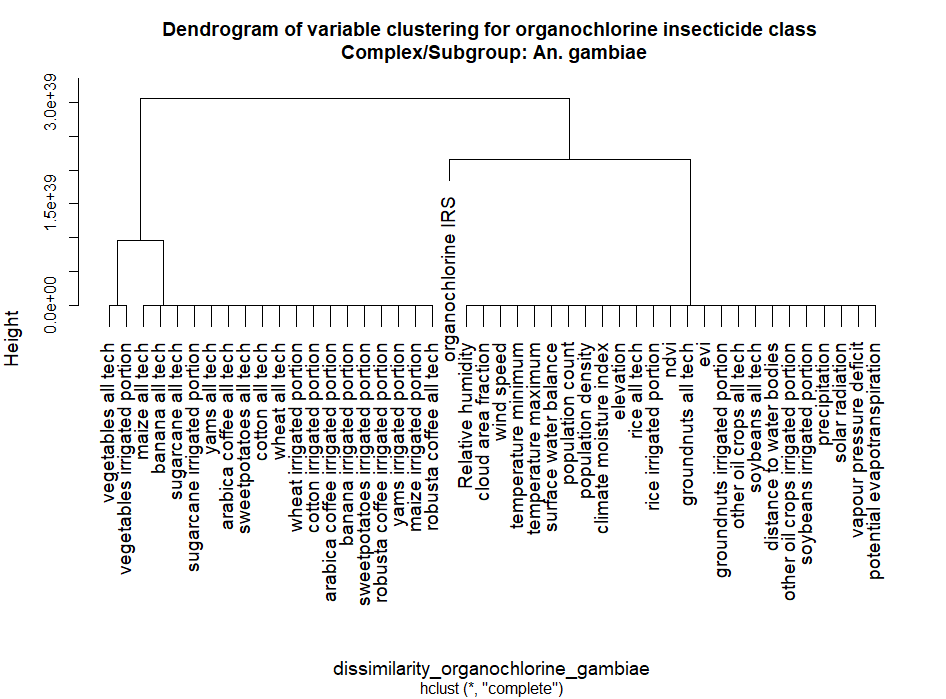
**

Figure S3

**
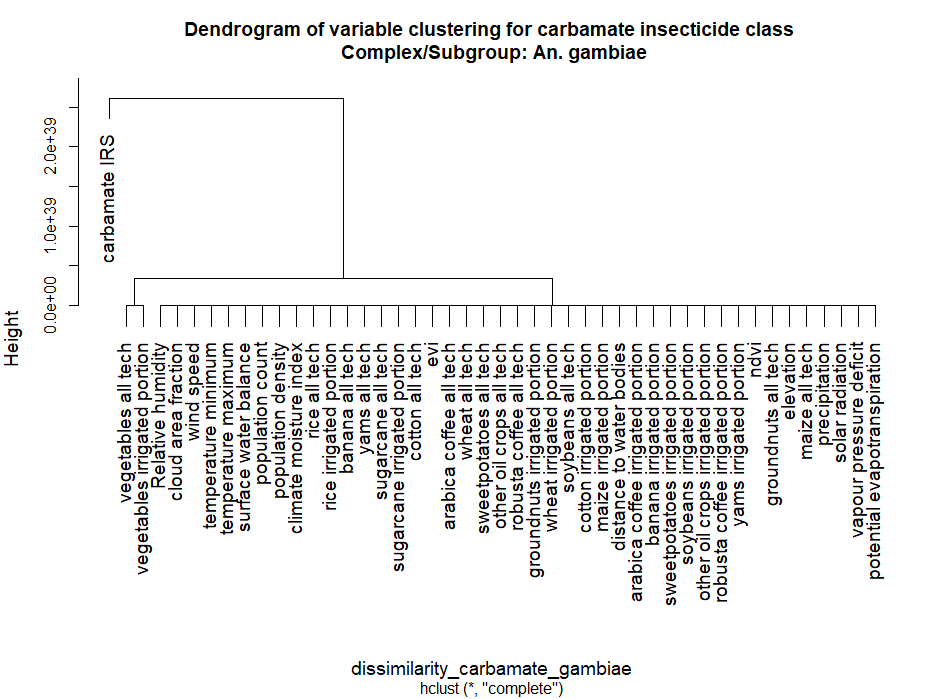
**

Figure S4

**
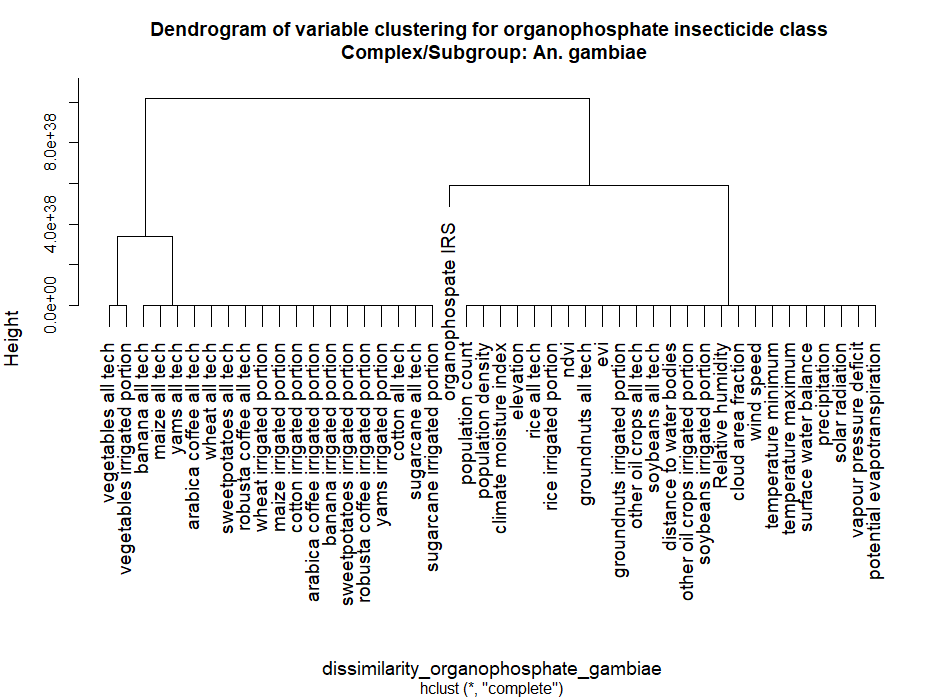
**

Figure S5


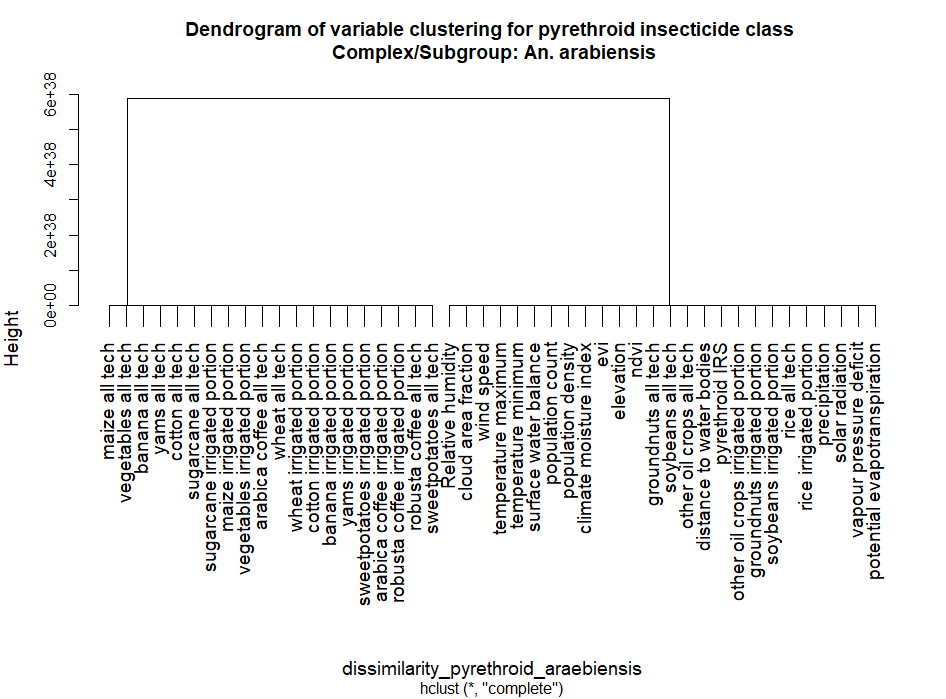


Figure S6


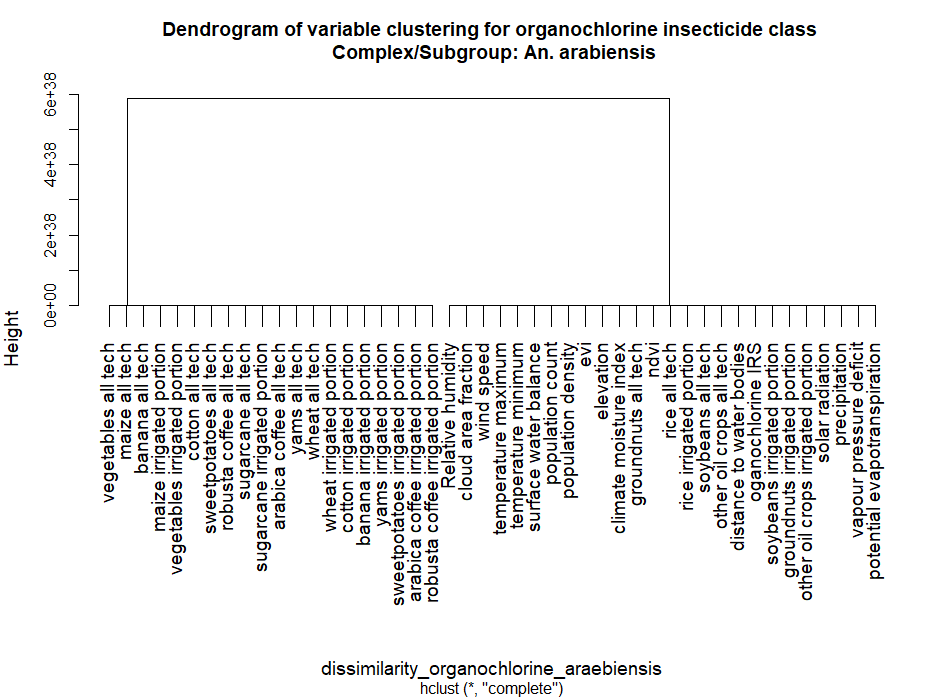


Figure S7


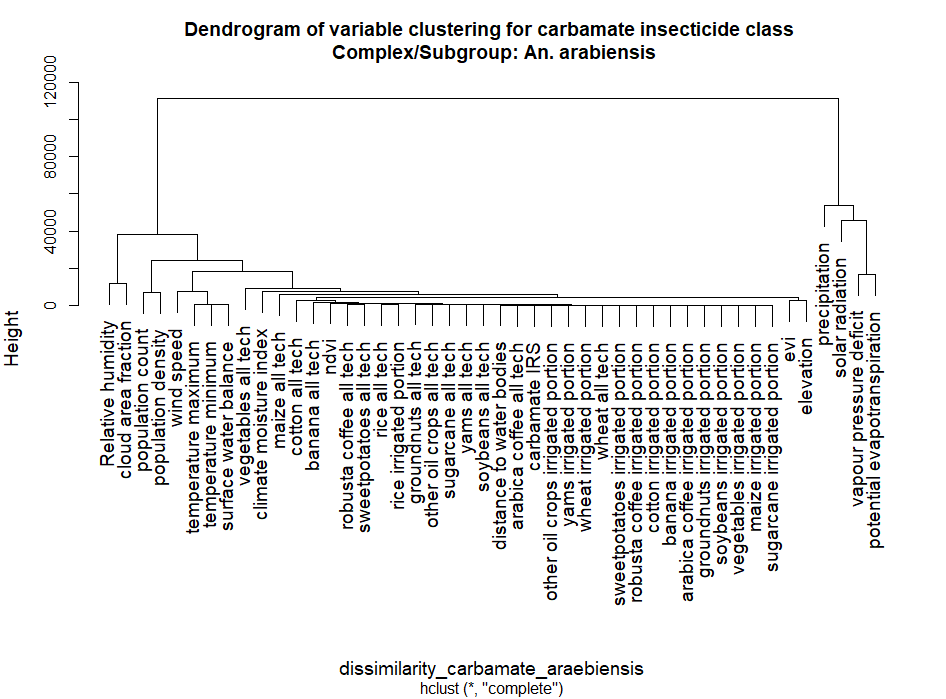


Figure S8


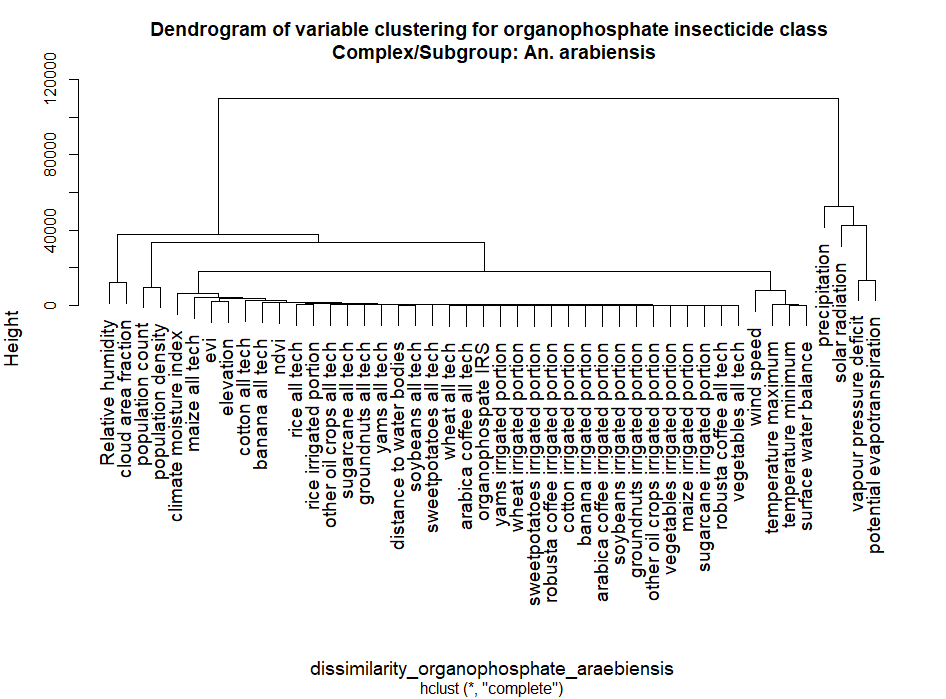


Figures S1-S8. FigS1- Variables clustering for pyrethroids insecticide class in *Anopheles gambiae* complex. FigS2 – Variables clustering for pyrethroids insecticide class in *Anopheles gambiae* complex FigS3 – Variables clustering for organochlorine insecticide class in *Anopheles gambiae* complex. FigS4 – Variables clustering for carbamate insecticide class in *Anopheles gambiae* complex. FigS5 – Variables clustering for organophosphate insecticide class in *Anopheles arabiensis.* FigS6 – Variables clustering for organochlorine insecticide class in *Anopheles arabiensis.* FigS7 – Variables clustering for carbamate insecticide class in *Anopheles arabiensis.* FigS8 – Variables clustering for organophosphate insecticide class in *Anopheles arabiensis.*
