# Supplementary material for: Spatio-temporal characterization of phenotypic resistance in malaria vector species
Source: BMC Biol. 2024 May 20;22:117. doi: 10.1186/s12915-024-01915-z (PMC11102860; doi:10.1186/s12915-024-01915-z)
Supplement: Supplementary file 4 — Additional file 4. Table S3. Chi square tests for associations between farming activities and confirmed insecticide resistance [file 12915_2024_1915_MOESM4_ESM.docx]

## Additional file 4: Table S3. Chi square tests for associations between farming activities and confirmed insecticide resistance

|  |  | *An. gambiae* complex | | | *Anopheles arabiensis* | | |
| --- | --- | --- | --- | --- | --- | --- | --- |
| Driver | Insecticide class | X-squared value | df | P-value | X-squared value | df | P-value |
| Rice farming | Pyrethroid | 10.326 | 2 | 0.0057 | 3.3440 | 2 | 0.1879 |
|  | Organochlorine | 1.5504 | 2 | 0.4606 | 15.0630 | 2 | 0.0005 |
|  | Carbamate | 65.305 | 2 | 0.0000 | 0.4906 | 2 | 0.7825 |
|  | Organophosphate | 7.9442 | 2 | 0.0188 | 1.1458 | 2 | 0.5639 |
| Rice irrigation | Pyrethroid | 29.168 | 2 | 0.0000 | 5.7154 | 2 | 0.0574 |
|  | Organochlorine | 13.656 | 2 | 0.0011 | 9.2255 | 2 | 0.0099 |
|  | Carbamate | 13.774 | 2 | 0.0010 | 1.7265 | 2 | 0.4218 |
|  | Organophosphate | 3.5401 | 2 | 0.1703 | 0.4507 | 2 | 0.7982 |
| Groundnuts farming | Pyrethroid | 35.965 | 2 | 0.0000 | 2.2137 | 2 | 0.3306 |
|  | Organochlorine | 13.717 | 2 | 0.0011 | 2.7461 | 2 | 0.2533 |
|  | Carbamate | 1.2469 | 2 | 0.5361 | 1.0083 | 2 | 0.6040 |
|  | Organophosphate | 32.711 | 2 | 0.0000 | 7.6412 | 2 | 0.0219 |
| Groundnuts irrigation | Pyrethroid | 28.038 | 2 | 0.0000 | - | - | - |
|  | Organochlorine | 4.2981 | 2 | 0.1166 | - | - | - |
|  | Carbamate | 28.006 | 2 | 0.0000 | - | - | - |
|  | Organophosphate | 2.255 | 2 | 0.3238 | - | - | - |
| Other oil crops farming | Pyrethroid | 7.9228 | 2 | 0.01904 | 8.8666 | 2 | 0.0119 |
|  | Organochlorine | 46.536 | 2 | 0.0000 | 15.1910 | 2 | 0.0005 |
|  | Carbamate | 5.1542 | 2 | 0.0760 | 3.2027 | 2 | 0.2016 |
|  | Organophosphate | 4.7204 | 2 | 0.0944 | 4.2188 | 2 | 0.1213 |
| Other oil crop irrigation | Pyrethroid | 18.271 | 2 | 0.0001 | 4.1145 | 2 | 0.1278 |
|  | Organochlorine | 4.9588 | 2 | 0.0837 | - | - | - |
|  | Carbamate | 11.9120 | 2 | 0.0025 | 4.2353 | 2 | 0.1203 |
|  | Organophosphate | 1.5701 | 2 | 0.4561 | - | - | - |
| Soybeans farming | Pyrethroid | 32.995 | 2 | 0.0000 | 7.7709 | 2 | 0.0205 |
|  | Organochlorine | 22.312 | 2 | 0.0001 | 8.2765 | 2 | 0.015605 |
|  | Carbamate | 83.522 | 2 | 0.0000 | 0.2898 | 2 | 0.8651 |
|  | Organophosphate | 3.2091 | 2 | 0.201 | 2.8759 | 2 | 0.2374 |
| Soybeans irrigation | Pyrethroid | 2.3524 | 2 | 0.3085 | - | - | - |
|  | Organochlorine | 0.63219 | 2 | 0.729 | - | - | - |
|  | Carbamate | 1.0721 | 2 | 0.5851 | - | - | - |
|  | Organophosphate | 2.8759 | 2 | 0.2374 | - | - | - |
| Arabica farming | Pyrethroid | 33.979 | 2 | 0.0000 | 5.1535 | 2 | 0.0760 |
|  | Organochlorine | 17.175 | 2 | 0.0002 | 0.4325 | 2 | 0.8055 |
|  | Carbamate | 62.682 | 2 | 0.0001 | 3.0545 | 2 | 0.2171 |
|  | Organophosphate | 3.5585 | 2 | 0.1688 | 0.62851 | 2 | 0.7303 |
| Arabica irrigation | Pyrethroid | 7.1398 | 2 | 0.02816 | - | - | - |
|  | Organochlorine | 2.4433 | 2 | 0.2948 | - | - | - |
|  | Carbamate | 13.71 | 2 | 0.0011 | - | - | - |
|  | Organophosphate | 1.4753 | 2 | 0.4782 | - | - | - |
| Banana farming | Pyrethroid | 37.729 | 2 | 0.0000 | 11.504 |  | 0.0032 |
|  | Organochlorine | 3.0478 | 2 | 0.2179 | 13.135 |  | 0.0014 |
|  | Carbamate | 34.876 | 2 | 0.0000 | 2.0158 |  | 0.365 |
|  | Organophosphate | 15.051 | 2 | 0.0005 | 1.6597 |  | 0.4361 |
| Banana irrigation | Pyrethroid | 3.996 | 2 | 0.1356 | 1.609 |  | 0.4473 |
|  | Organochlorine | 6.1814 | 2 | 0.04547 | 6.8721 |  | 0.03219 |
|  | Carbamate | 10.437 | 2 | 0.0054 | - | - | - |
|  | Organophosphate | 0.0245 | 2 | 0.9878 | - | - | - |
| Cotton farming | Pyrethroid | 3.768 | 2 | 0.152 | 8.6777 |  | 0.01305 |
|  | Organochlorine | 5.44 | 2 | 0.06587 | 9.1677 |  | 0.01022 |
|  | Carbamate | 26.255 | 2 | 0.0000 | 2.7624 |  | 0.2513 |
|  | Organophosphate | 2.3333 | 2 | 0.3114 | 3.3914 |  | 0.1835 |
| Cotton irrigation | Pyrethroid | 95.205 | 2 | 0.0000 | 11.423 |  | 0.0033 |
|  | Organochlorine | 6.69 | 2 | 0.03526 | 14.581 |  | 0.0007 |
|  | Carbamate | 59.472 | 2 | 0.0000 | - | - | - |
|  | Organophosphate | 2.5279 | 2 | 0.2825 | - | - | - |
| Maize farming | Pyrethroid | 14.849 | 2 | 0.0006 | 7.9974 |  | 0.01834 |
|  | Organochlorine | 0.5366 | 2 | 0.7647 | 2.5284 |  | 0.2825 |
|  | Carbamate | 20.649 | 2 | 0.0000 | 3.0545 |  | 0.2171 |
|  | Organophosphate | 8.8593 | 2 | 0.0119 | 1.0478 |  | 0.5922 |
| Maize irrigation | Pyrethroid | 4.9908 | 2 | 0.08246 | 1.3138 |  | 0.5185 |
|  | Organochlorine | 9.4322 | 2 | 0.00895 | 7.7659 |  | 0.02059 |
|  | Carbamate | 10.849 | 2 | 0.0044 | 3.3277 |  | 0.1894 |
|  | Organophosphate | 0.23948 | 2 | 0.8871 | 3.5716 |  | 0.1677 |
| Robusta farming | Pyrethroid | 52.817 | 2 | 0.0044 | 9.8578 |  | 0.0072 |
|  | Organochlorine | 5.4197 | 2 | 0.06655 | 11.54 |  | 0.00312 |
|  | Carbamate | 4.0216 | 2 | 0.1339 | 16.978 |  | 0.0002 |
|  | Organophosphate | 0.89651 | 2 | 0.6387 | 0.9722 |  | 0.615 |
| Robusta irrigation | Pyrethroid | 3431.4 | 2 | 0.0000 | 89.495 |  | 0.0000 |
|  | Organochlorine | 849.3 | 2 | 0.0000 | 13.153 |  | 0.0014 |
|  | Carbamate | 601.36 | 2 | 0.0000 | 0.6667 |  | 0.7165 |
|  | Organophosphate | 1296.7 | 2 | 0.0000 | 30.229 |  | 0.0000 |
| Sugarcane farming | Pyrethroid | 63.463 | 2 | 0.0000 | 2.3905 |  | 0.3026 |
|  | Organochlorine | 3.2371 | 2 | 0.1982 | 6.2922 |  | 0.04302 |
|  | Carbamate | 4.6835 | 2 | 0.09616 | 7.2894 |  | 0.02613 |
|  | Organophosphate | 0.5781 | 2 | 0.749 | 0.0028 |  | 0.9986 |
| Sugarcane irrigation | Pyrethroid | 68.042 | 2 | 0.0000 | 6.2948 |  | 0.04296 |
|  | Organochlorine | 1.2744 | 2 | 0.5288 | 7.6738 |  | 0.02156 |
|  | Carbamate | 6.1733 | 2 | 0.04565 | 2.3558 |  | 0.3079 |
|  | Organophosphate | 7.4145 | 2 | 0.02455 | 0.9722 |  | 0.615 |
| Sweet potatoes farming | Pyrethroid | 38.856 | 2 | 0.0000 | 2.5203 |  | 0.2836 |
|  | Organochlorine | 18.668 | 2 | 0.0000 | 9.469 |  | 0.0087 |
|  | Carbamate | 6.3394 | 2 | 0.04202 | 1.7679 |  | 0.4132 |
|  | Organophosphate | 11.183 | 2 | 0.003729 | 5.3091 |  | 0.07033 |
| Sweet potatoes irrigation | Pyrethroid | 5.0087 | 2 | 0.0817 | - | - | - |
|  | Organochlorine | 10.434 | 2 | 0.0054 | - | - | - |
|  | Carbamate | 12.605 | 2 | 0.0018 | - | - | - |
|  | Organophosphate | 5.5812 | 2 | 0.0614 | - | - | - |
| Vegetables farming | Pyrethroid | 17.271 | 2 | 0.0002 | 2.5203 |  | 0.2836 |
|  | Organochlorine | 28.398 | 2 | 0.0000 | 9.469 |  | 0.0088 |
|  | Carbamate | 37.981 | 2 | 0.0000 | 1.7679 |  | 0.4132 |
|  | Organophosphate | 11.328 | 2 | 0.0034 | 5.3091 |  | 0.0703 |
| Vegetables irrigation | Pyrethroid | 5.0087 | 2 | 0.08173 | - | - | - |
|  | Organochlorine | 10.434 | 2 | 0.0054 | - | - | - |
|  | Carbamate | 12.605 | 2 | 0.0018 | - | - | - |
|  | Organophosphate | 5.5812 | 2 | 0.0614 | - | - | - |
| Wheat farming | Pyrethroid | 391.12 | 2 | 0.0000 | 17.859 |  | 0.0001 |
|  | Organochlorine | 43.115 | 2 | 0.0000 | 14.458 |  | 0.0007 |
|  | Carbamate | 124.15 | 2 | 0.0000 | - | - | - |
|  | Organophosphate | 1.4249 | 2 | 0.4904 | 0.30501 |  | 0.8586 |
| Wheat irrigation | Pyrethroid | 33.63 | 2 | 0.0000 | 24.294 |  | 0.0001 |
|  | Organochlorine | 5.749 | 2 | 0.0564 | 14.581 |  | 0.000682 |
|  | Carbamate | 31.626 | 2 | 0.0564 | - | - | - |
|  | Organophosphate | 10.537 | 2 | 0.0051 | - | - | - |
| Yams farming | Pyrethroid | 136.04 | 2 | 0.0000 | 28.747 |  | 0.0001 |
|  | Organochlorine | 99.188 | 2 | 0.0000 | 16.928 |  | 0.0002 |
|  | Carbamate | 190 | 2 | 0.0000 | 12.587 |  | 0.0018 |
|  | Organophosphate | 21.578 | 2 | 0.0000 | 0.65495 |  | 0.7207 |
| Yams irrigation | Pyrethroid | 3431.4 | 2 | 0.0000 | 89.495 |  | 0.0001 |
|  | Organochlorine | 849.3 | 2 | 0.0000 | 13.153 |  | 0.001393 |
|  | Carbamate | 601.36 | 2 | 0.0000 | 0.66667 |  | 0.7165 |
|  | Organophosphate | 1296.7 | 2 | 0.0000 | 30.229 |  | 0.0001 |
