# Supplementary material for: Spatio-temporal characterization of phenotypic resistance in malaria vector species
Source: BMC Biol. 2024 May 20;22:117. doi: 10.1186/s12915-024-01915-z (PMC11102860; doi:10.1186/s12915-024-01915-z)
Supplement: Supplementary file 5 — Additional file 5. Table S4. Comparisons between cellular automata (CA) models’ outputs and actual confirmed IR state in An. gambiae complex [file 12915_2024_1915_MOESM5_ESM.docx]

## Additional file 5: Table S4. Comparisons between cellular automata (CA) models’ outputs and actual confirmed IR state in An. gambiae complex

|  | **Accuracies attained on fine-tuning cellular automata model for confirmed IR in *Anopheles gambiae* complex** | | | | | | | | | | |
| --- | --- | --- | --- | --- | --- | --- | --- | --- | --- | --- | --- |
|  | **Ethiopia** | | | | **Cameroon** | | | **Burkina Faso** | | | |
| **Year** | **Pyrethroid** | **Organochlorine** | **Carbamate** | **Organophosphate** | **Pyrethroid** | **Organochlorine** | **Carbamate** | **Pyrethroid** | **Organochlorine** | **Carbamate** | **Organophosphate** |
| 2001 | - | - | - | - | 0.0000 |  | - | - | 1.0000 |  | - |
| 2002 | - | - | - | - | 1.0000 |  | - | - | - |  | - |
| 2003 | - | - | - | - | 1.0000 |  | - | - | - |  | - |
| 2004 | - | - | - | - | - |  | - | - | - |  | - |
| 2005 | - | 0.6667 | - | - | 0.6667 | 0.7500 | - | - | - |  | - |
| 2006 | - | 1.0000 | - | - |  | 1.0000 | - | 1.0000 | 0.7500 |  | - |
| 2007 | 0.5000 | 1.0000 | - | - | 0.8333 | 0.8333 | - |  | - |  | - |
| 2008 | 0.8571 | 0.7500 | - | 0.0000 |  | - | - | 0.8140 | 0.8750 | 0.7727 | - |
| 2009 | 0.8462 | 0.8750 | - | 1.0000 |  | - | - | 0.8788 | 0.9583 | 0.7368 | 1.0000 |
| 2010 | 1.0000 | 0.8750 | - | 1.0000 |  | - | 1.0000 | 0.9394 | 1.0000 | 1.0000 | 1.0000 |
| 2011 | 0.3333 | 0.8000 | - | 1.0000 | 1.0000 | - |  | 1.0000 | 1.0000 | 1.0000 | 1.0000 |
| 2012 | 0.8846 | 0.8750 | 1.0000 | 0.8000 | 1.0000 | 1.0000 | 1.0000 | 1.0000 | 1.0000 | 1.0000 | - |
| 2013 | 0.9333 | 0.8000 | 0.5000 | 0.8667 | 0.9091 | 1.0000 | 1.0000 | 1.0000 | 1.0000 | 1.0000 | 1.0000 |
| 2014 | 0.9342 | 0.9062 | 0.6667 | 0.6200 | 0.9333 | 1.0000 | - | - | - |  | - |
| 2015 | 0.9487 | 0.7727 | 1.0000 | 0.7500 | 0.9667 | - | - | 1.0000 | 1.0000 | 1.0000 | - |
| 2016 | 0.8889 | 0.8000 | 1.0000 | 0.8000 | - | - | - | - | - |  | - |
| 2017 | 0.8411 | 0.7000 | - | - | - | - | - | - | - |  | - |
| **Mean accuracy** | **0.8047** | **0.8320** | **0.8333** | **0.7596** | **0.8092** | **0.9306** | **1.0000** | **0.9540** | **0.9537** | **0.9299** | **1.0000** |
|  | **Accuracies attained following cellular automata model validation** | | | | | | |  |  |  |  |
|  | **Nigeria** | | | | **Uganda** | | |  |  |  |  |
| 2001 | 1.0000 | - | - | - | 1.0000 | - | - | - | - | - | - |
| 2002 | 0.0000 | 0.7500 | - | - | - | - | - | - | - | - | - |
| 2003 | - | 1.0000 | - | - | - | - | - | - | - | - | - |
| 2004 | - | 1.0000 | - | - | 1.0000 | - | - | - | - | - | - |
| 2005 | - | 1.0000 | - | - | 0.0000 | 1.0000 | - | - | - | - | - |
| 2006 | - | - | - | - | 0.6250 | 0.8750 | - | - | - | - | - |
| 2007 | - | - | - | - | 0.8333 | - | - | - | - | - | - |
| 2008 | - | - | - | - | - | 0.8333 | - | - | - | - | - |
| 2009 | 0.0000 | 1.0000 | - | - | - | - | - | - | - | - | - |
| 2010 | 0.5000 | 0.9091 | 0.4200 | - | - | - | - | - | - | - | - |
| 2011 | - | - | - | - | 1.0000 | 1.0000 | - | - | - | - | - |
| 2012 | - | - | - | - | 1.0000 | 1.0000 |  | - | - | - | - |
| 2013 | 1.0000 | 1.0000 | - | - |  | - | - | - | - | - | - |
| 2014 | 0.7333 | 1.0000 | - | - |  | - | - | - | - | - | - |
| 2015 | 1.0000 | 1.0000 | 5.0000 | 1.0000 | 1.0000 | - | - | - | - | - | - |
| 2016 | 0.9756 | 1.0000 | 1.0000 | 1.0000 | 0.5000 | - | - | - | - | - | - |
| 2017 | 0.8790 | 1.0000 | 1.0000 | 1.0000 | 0.8966 | 1.0000 | 1.0000 | - | - | - | - |
| **Mean accuracy** | **0.7704** | **0.8442** | **0.8750** | **1.0000** | **0.7855** | **0.9514** | **1.0000** | - | - | - | - |

CA – cellular automata; - no actual data on confirmed insecticide resistance (IR
