# Supplementary material for: Spatio-temporal characterization of phenotypic resistance in malaria vector species
Source: BMC Biol. 2024 May 20;22:117. doi: 10.1186/s12915-024-01915-z (PMC11102860; doi:10.1186/s12915-024-01915-z)
Supplement: Supplementary file 6 — Additional file 6. Table S5. Data sources [47–53]. [file 12915_2024_1915_MOESM6_ESM.docx]

## Additional file [6](https://parasitesandvectors.biomedcentral.com/articles/10.1186/s13071-017-2342-y#MOESM1): Table S5. Data sources

| Variable | Data source link | Spatial resolution in kilometers | Data citation |
| --- | --- | --- | --- |
| Precipitation | https://www.envidat.ch/#/metadata/bioclim_plus | 1.0000 | [47] |
| Temperature Max | https://www.envidat.ch/#/metadata/bioclim_plus | 1.0000 | [47] |
| Temperature Min | https://www.envidat.ch/#/metadata/bioclim_plus | 1.0000 | [47] |
| Relative humidity | https://www.envidat.ch/#/metadata/bioclim_plus | 1.0000 | [47] |
| Wind speed | https://www.envidat.ch/#/metadata/bioclim_plus | 1.0000 | [47] |
| Solar Radiation | https://www.envidat.ch/#/metadata/bioclim_plus | 1.0000 | [47] |
| Cloud Area Fraction | https://www.envidat.ch/#/metadata/bioclim_plus | 1.0000 | [47] |
| Vapour Pressure Deficit | https://www.envidat.ch/#/metadata/bioclim_plus | 1.0000 | [47] |
| Potential Evapotranspiration | https://www.envidat.ch/#/metadata/bioclim_plus | 1.0000 | [47] |
| Climate Moisture Index | https://www.envidat.ch/#/metadata/bioclim_plus | 1.0000 | [47] |
| Site Water Balance | https://www.envidat.ch/#/metadata/bioclim_plus | 1.0000 | [47] |
| EVI | https://developers.google.com/earth-engine/datasets/catalog/MODIS_061_MOD13A2 | 1.0000 | [48] |
| NDVI | https://developers.google.com/earth-engine/datasets/catalog/MODIS_061_MOD13A2 | 1.0000 | [48] |
| LULC (LC_Type1) | https://developers.google.com/earth-engine/datasets/catalog/MODIS_061_MCD12Q1#description | 0.5000 | [49] |
| Elevation | https://developers.google.com/earth-engine/datasets/catalog/USGS_GMTED2010#description | 0.2319 | [50] |
| Distance to Water bodies | https://catalogue.ceda.ac.uk/uuid/84d4f66b668241328df0c43f8f3b3e16?jump=related-docs-anchor | 0.3000 | [51] |
| Crop (Physical Area) | https://www.ifpri.org/project/harvestchoice | 1.0000 | [52] |
| Population count | https://developers.google.com/earth-engine/datasets/catalog/CIESIN_GPWv411_GPW_Population_Count#description | 0.9277 | [53] |
| Population Density | https://developers.google.com/earth-engine/datasets/catalog/CIESIN_GPWv411_GPW_Population_Density | 0.9277 | [53] |
